# Paving the way toward highly reversible aluminum metal anodes: the core role of ionic liquid electrolyte systems

**DOI:** 10.1039/d6gc03584a

**Published:** 2026-09-25

**Authors:** Huaming Yu, Dongping Chen, Tingrui Yang, Xiaofeng Zhang, Minshen Zhu, Wenjun Meng, Yang Huang

**Affiliations:** a Advanced Materials Thrust, Function Hub, The Hong Kong University of Science and Technology (Guangzhou) Guangzhou 511400 P. R. China yanghuang@hkust-gz.edu.cn; b MIIT Key Laboratory of Critical Materials Technology for New Energy Conversion and Storage, School of Chemistry and Chemical Engineering, Harbin Institute of Technology Harbin 150000 P. R. China; c Research Center for Materials, Architectures, and Integration of Nanomembranes (MAIN), TU Chemnitz Chemnitz 09126 Germany minshen.zhu@main.tu-chemnitz.de; d Guangdong Provincial Key Laboratory of Efficient Catalysis and Energy Conversion, Beijing Institute of Technology Zhuhai Zhuhai 519088 P. R. China mengwenjun@bitzh.edu.cn

## Abstract

Rechargeable aluminum-ion batteries (AIBs) are promising candidates for next-generation energy storage, yet their practical deployment is hampered by the intrinsic limitations of conventional ionic liquid (IL) electrolytes, such as high viscosity, corrosivity, and moisture sensitivity. This review focuses on the core role of IL electrolyte design in enabling highly reversible aluminum metal anodes. First, the fundamental requirements and working mechanisms of ILs are systematically elucidated. Furthermore, recent optimization strategies are critically summarized and discussed in terms of four aspects: (1) cation–anion pair engineering to regulate active aluminum species, tailor physicochemical properties, and reduce reliance on costly imidazolium-based components, (2) functional additives to enhance ion transport and regulate solid electrolyte interphase (SEI) formation, (3) deep eutectic solvent formulations as potentially lower-cost and more sustainable alternatives, and (4) gel and quasi-solid-state architecture construction to improve safety and mechanical strength. Finally, the performance–sustainability trade-offs of current IL electrolyte systems are discussed, and design guidelines are provided for developing safer and more sustainable electrolytes with high ionic conductivity, wide electrochemical windows, and robust interfacial compatibility. These advancements are expected to facilitate the realization of highly reversible aluminum metal anodes and high-energy-density AIBs, thereby propelling the development of next-generation aluminum-based energy storage technologies.

## Abstract 2

This review highlights ionic liquid electrolytes for reversible Al anodes and summarizes four strategies—cation–anion engineering, additives, deep eutectic solvents, and gel electrolytes—for safe, low-cost, high-performance Al-ion batteries.

## Graphical abstract

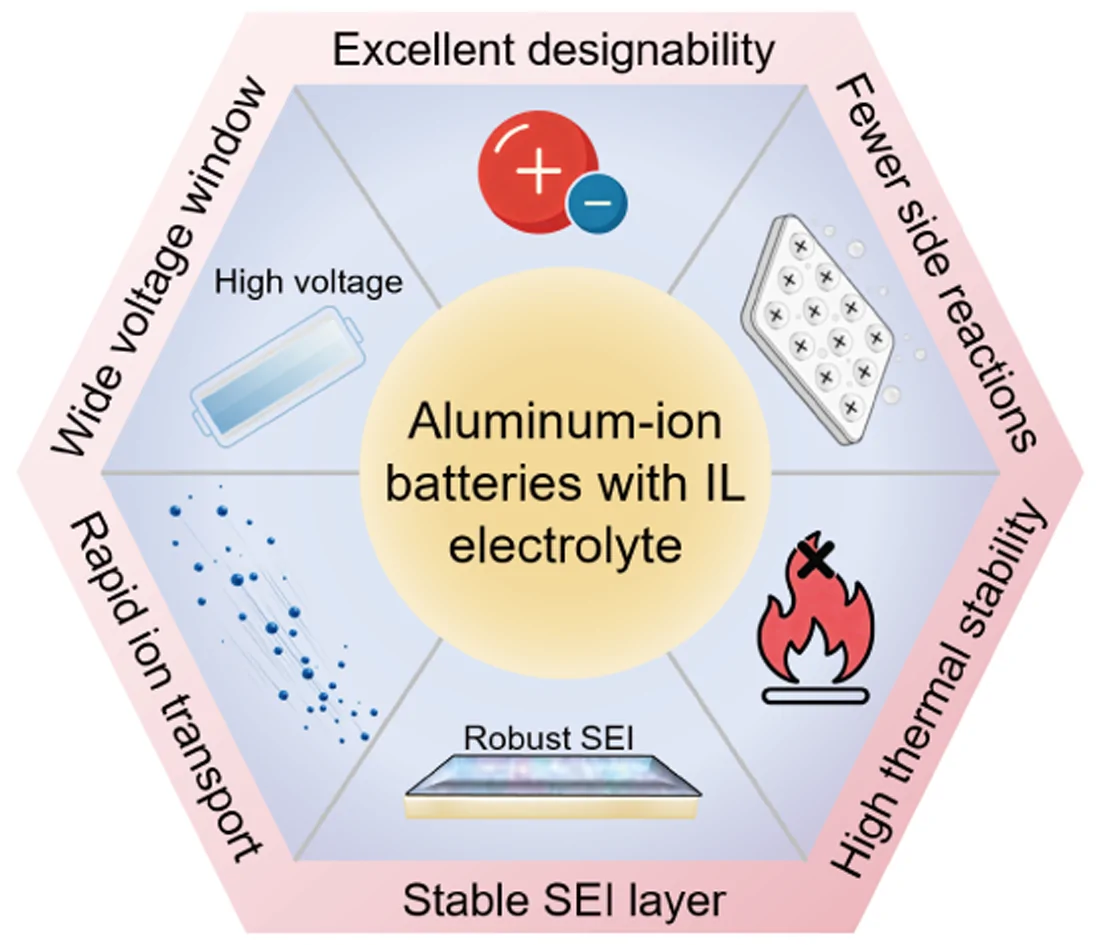

**Green foundation**

1. Advanced ionic liquid (IL) electrolyte design improves the safety and sustainability of rechargeable aluminum batteries by reducing flammability, improving electrolyte utilization, and lowering electrolyte cost. This review summarizes four complementary strategies: cation–anion engineering, functional additives, deep eutectic solvent formulations, and gel polymer electrolytes toward more sustainable IL systems.

2. Improved IL electrolytes enable highly reversible aluminum deposition/stripping while enhancing battery safety and sustainability. Coupled with earth-abundant aluminum anodes, they provide a promising route toward low-carbon and resource-efficient energy storage for large-scale applications.

3. This review highlights the trade-offs among electrochemical performance, safety, cost, and sustainability, and proposes design guidelines for balancing these competing requirements. These insights provide a framework for developing safer and more sustainable IL electrolytes for next-generation aluminum-ion batteries.

## 1. Introduction

Electrochemical energy storage systems are indispensable to the development of portable electronics, electric vehicles, and smart grids.^1,2^ While lithium-ion batteries (LIBs) have achieved remarkable technological and commercial success over the past two decades, their continued dominance is challenged by the escalating demand for higher energy densities. Furthermore, LIBs face several critical limitations, including supply-chain vulnerabilities caused by increasing lithium consumption, volatile raw-material costs, and serious safety risks associated with dendrite formation.^3,4^ Consequently, the development of alternative energy storage systems with high capacity, low cost, sustainability, and intrinsic safety has become a major priority for both academia and industry.^5–7^ In this context, aluminum-ion batteries (AIBs) have emerged as a highly promising candidate.

**Fig. 1:**
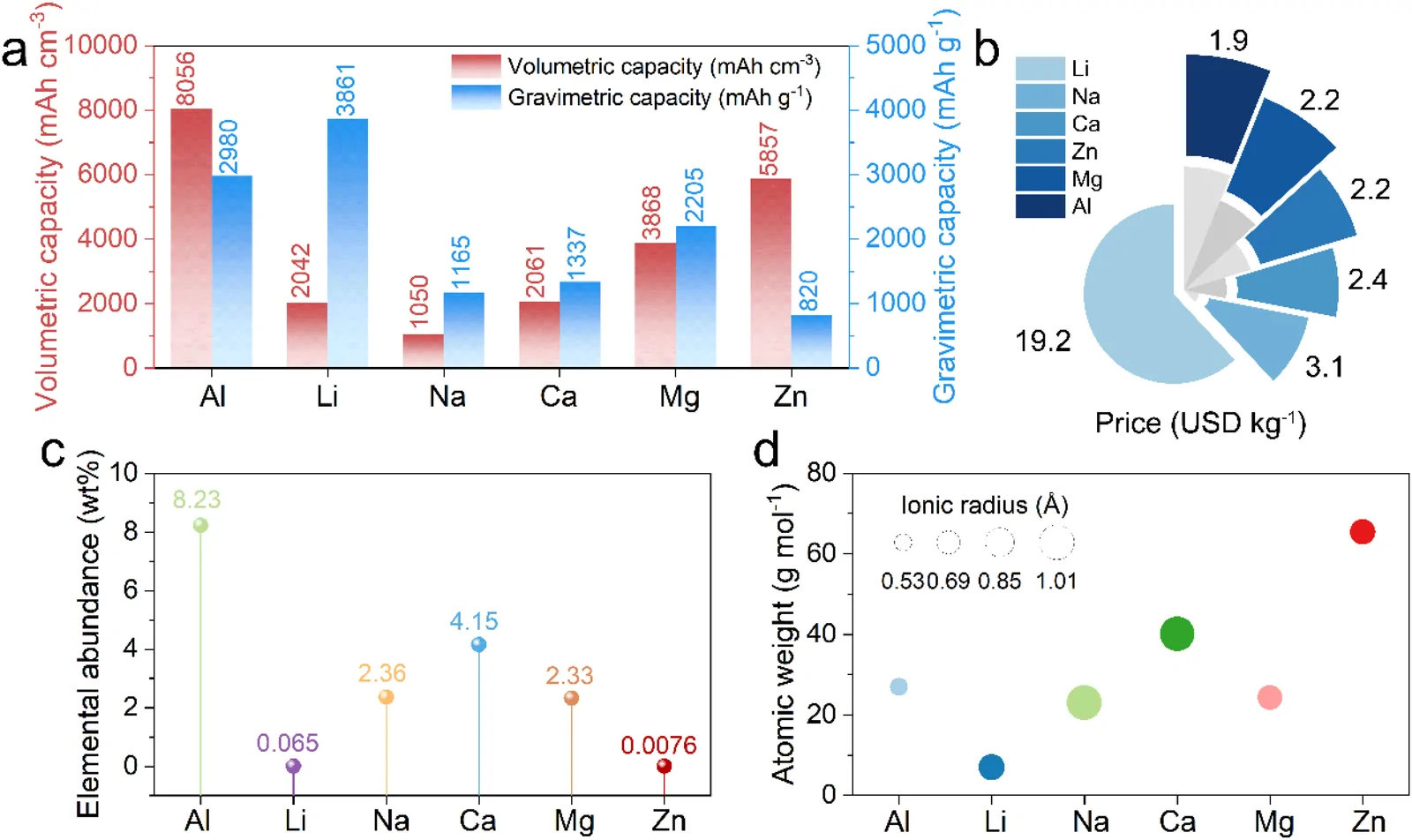
Comparison between (a) volumetric and gravimetric capacities, (b) cost of different metal, (c) elemental abundance, (d) atomic weight and ionic radius in different electrochemical systems.

Aluminum offers distinct advantages owing to its natural abundance, low cost, low density, and high theoretical capacity (Fig. 1a–c). As the most abundant metal in the Earth's crust, accounting for approximately 8.2 wt%, and the third most abundant element overall, Al provides a clear economic advantage over many other battery-relevant metals.^8^ Furthermore, its relatively high electronegativity ensures lower reactivity in ambient air and moisture, inherently boosting safety.^9,10^ Electrochemically, three-electron redox reaction of aluminum yields a theoretical volumetric capacity of 8056 mAh cm^−3^, nearly four times that of lithium (2042 mAh cm^−3^). Its theoretical gravimetric capacity is 2980 mAh g^−1^, second only to lithium among commonly studied metal anodes.^11,12^ In addition, the small ionic radius of Al^3+^ (0.535 Å) could facilitate favorable intercalation dynamics (Fig. 1d). Therefore, aluminum foil anodes present a compelling platform for next-generation energy storage.

The development of AIBs has spanned more than a century, marked by repeated technological explorations, breakthroughs of bottlenecks, and continuous optimization. The earliest AIBs can be traced back to 1859, when Hulot designed a basic electrochemical cell that employed zinc or mercury as the anode, aluminum as the cathode, and a dilute aqueous sulfuric acid solution as the electrolyte.^13,14^ In 1948, Heise *et al.* developed a heavy-duty chlorine-depolarized cell that employed aluminum as the anode, with an operating voltage of 2.25–2.45 V.^15^ In 1972, Holleck reported an aluminum/chlorine battery based on a molten salt electrolyte, which resembled an aluminum/air battery system and employed a carbon cathode as a gas diffusion electrode to facilitate reactions at the solid–liquid–gas three-phase interface.^16^ In the 1980s, the combination of sulfide cathodes and molten salt electrolytes laid the foundation for the early development of AIBs. However, their practical application was constrained by the high operating temperature required for molten salt electrolytes and by chlorine gas evolution.^17,18^ It was not until 1985 that the introduction of room-temperature ionic liquid (IL) electrolytes superseded high-temperature molten salts, enabling the reversible plating and stripping of aluminum at ambient temperatures.^19^ Over the following three decades, numerous researchers have consistently explored the design and application of novel organic IL systems for rechargeable aluminum batteries, aiming to enhance their electrochemical performance.^20–22^ A major milestone was reached in 2015, when Lin *et al.* reported an ultrafast and long-life AIB utilizing a 3D graphitic foam cathode and an AlCl_3_/[EMIm]Cl IL electrolyte.^23^ This pioneering work laid a crucial foundation for IL electrolytes in room-temperature AIBs and stimulated broad interest in this field.

Electrolyte development has played a critical role in the historical progression of AIBs. Among the reported systems, AlCl_3_/[EMIm]Cl has been recognized as a standout electrolyte for AIBs, primarily owing to its outstanding performance metrics and room-temperature viability^24^ A major drawback of the AlCl_3_/[EMIm]Cl electrolyte is its reliance on chloroaluminate species (like AlCl_4_^−^ and Al_2_Cl_7_^−^) as charge carriers rather than bare Al^3+^, which inherently limits the attainable theoretical capacity. Second, the abundant presence of Al_2_Cl_7_^−^ imparts strong corrosivity to the system, vigorously attacking common metallic components, including aluminum, titanium, nickel, and iron. This corrosivity places strict constraints on the selection of cell casings and current collectors.^25–28^ Crucially, the inherent moisture sensitivity and high cost of conventional IL electrolytes impose significant hurdles to their commercial deployment. To address these issues, recent research has focused on modifying the AlCl_3_/[EMIm]Cl system through several approaches, including replacing aluminum chloride with alternative salts to reduce corrosivity, using low-cost organic ligands to decrease electrolyte cost, incorporating functional additives to improve interfacial stability, and developing gel or quasi-solid state electrolytes to enhance overall cell stability.^29–35^ Despite these advances, the intrinsic limitations of AlCl_3_/[EMIm]Cl electrolytes have not yet been fully resolved.

Current investigations into IL electrolytes for AIBs remain predominantly exploratory. Therefore, there is still a need to rationally engineer stable, efficient, and cost-effective electrolyte systems for practical applications. Although several reviews have discussed AIBs and related electrolytes, a focused and critical assessment of IL electrolyte architecture remains limited. In this review, we focus on IL electrolytes and discuss their fundamental requirements and working mechanisms in enabling reversible aluminum metal anodes. We then summarize recent breakthroughs and strategies of electrolyte optimization, including composition regulation, additive engineering, deep eutectic electrolyte design, and gel or quasi-solid-state electrolyte construction. Finally, the remaining challenges and future directions for developing high-performance IL electrolyte systems for next-generation aluminum-based energy storage are discussed.

## 2. Ionic liquid electrolytes: unique properties and key functions

The working principle of rechargeable AIBs is founded upon the reversible plating/stripping of the aluminum layer during charge and discharge cycles.^36,37^ In a manner consistent with other metal-ion systems, the electrolyte in rechargeable AIBs acts as the conductive medium for ion shuttle between electrodes, governing the reversible plating of the aluminum anode. Since the electrolyte composition fundamentally governs key parameters such as capacity, stability, and safety, the selection process must be rigorously controlled.^38–40^

Based on the choice of electrolyte solvent, AIBs are categorized into aqueous and non-aqueous systems. Although aqueous electrolytes possess high ionic conductivity and inherent safety, their narrow electrochemical stability window and low redox potential often trigger detrimental side reactions at the anode, such as self-corrosion, irreversible hydrogen evolution, and the formation of an aluminum hydroxide passivation layer. These parasitic processes severely undermine reversible aluminum deposition, reduce coulombic efficiency (CE), and destabilize the electrode/electrolyte interface, ultimately limiting the energy density and cycle lifespan of aqueous AIBs.^41–44^ By contrast, the electrochemical potential of aluminum can be remarkably augmented through the deployment of non-aqueous electrolytes, which predominantly consist of ILs, eutectic mixtures acting as IL analogues, and IL-hybridized quasi-solid-state matrices.^22,45–47^ By transcending the innate bottlenecks of aqueous media, these electrolytes successfully realize highly reversible anodic plating/stripping while maintaining a broad electrochemical window (≥2.5 V). This expanded operating range is crucial for accommodating advanced high-capacity cathode materials, such as graphite, porphyrins, and various sulfides or organic frameworks, thereby enhancing the overall energy density of the system.^48^ Moreover, the capacity of these electrolytes to enable rapid AlCl_4_^−^ intercalation is indispensable for maintaining the cyclic integrity of AIBs. Such efficient ion-insertion behavior is particularly vital for preserving performance stability during high-current-density operation, ensuring robust long-term reversibility.

Frequently referred to as room-temperature molten salts, ILs fundamentally consist of long-chain organic cations paired with either organic or inorganic anions.^49^ ILs exhibit suppressed melting points below environmental temperatures, a phenomenon arising from the combination of weak inter-cationic separation and shielded electrostatic forces. By definition, this structural configuration classifies them as ionic media that remain in a fluid state under ambient conditions.^24^ Despite fragmented reports appearing as early as the 1800s, ILs did not gain substantial prominence in the scientific canon until the twilight of the 20^th^ century. Subsequently, the field witnessed a rapid increase in annual publications, encompassing tens of thousands of studies across hundreds of disciplines. Such a monumental surge has established ionic liquids as a key research focus of ILs as a vital frontier in modern chemistry.

**Fig. 2:**
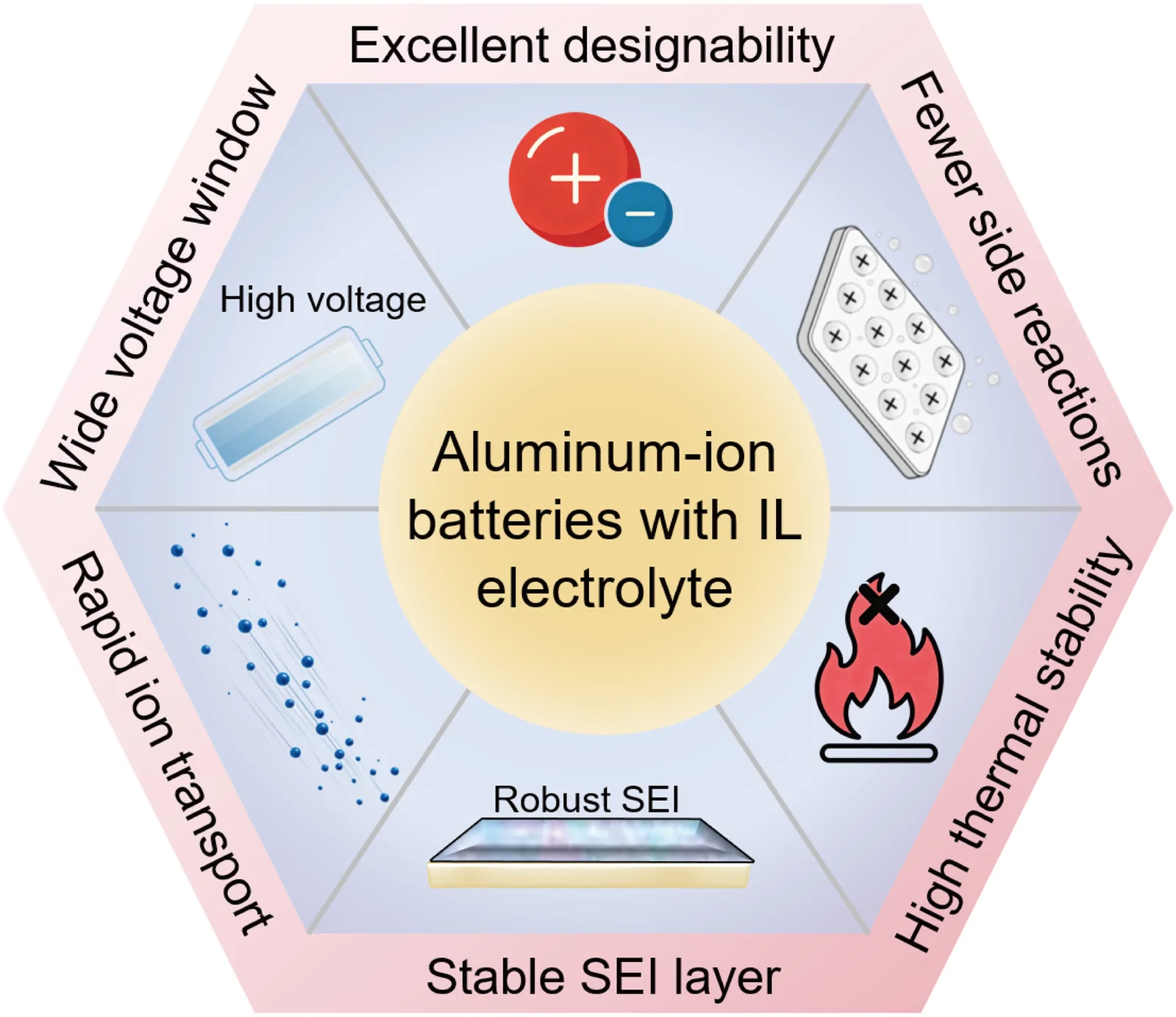
Functionalities and features of ILs in AIBs.

A pivotal advantage of ILs lies in their constitution of discrete cationic and anionic species, which confers highly tunable and favorable characteristics. Consequently, the intrinsic physicochemical properties of these electrolyte systems are readily adjustable *via* structural modifications, such as tuning the functional group symmetry or altering the carbon chain length^50–53^ Additionally, owing to their classification as environmentally friendly green solvents, ILs exhibit robust capabilities for dissolving a broad spectrum of organic and inorganic salt species.^54^ By serving as electrolytes for metal batteries, ILs offer a rich palette of tunable features that enhance overall electrochemical efficiency (Fig. 2). More precisely, the incorporation of specific functional groups into IL architectures allows for the regulation of metal-ion solvation shells and electrolyte hydrogen-bonding arrays, ultimately extending electrochemical stability and promoting superior ionic kinetics.^55–57^ Concurrently, many IL electrolytes can form a stable SEI layer on the electrode surface to suppress side reactions and uncontrollable dendrite growth in the system.^58,59^ IL electrolytes can also impart non-flammability to the battery or prevent the electrolyte from freezing, thereby ensuring the stable exertion of electrochemical performance in extreme temperature environments.^60^ Furthermore, due to the high affinity between ILs and rigid polymer backbones, ILs can functionally modify gel polymer electrolytes or solid polymer electrolytes to enhance material mechanical strength and ion transport.^61–64^ Overall, IL electrolytes possess excellent chemical stability, thermal stability, a wide electrochemical window, extremely low flammability and volatility, as well as high ionic conductivity, which gives them broad application prospects in the field of electrolytes for AIBs.^65,66^

Electrolytes based on chloroaluminate ILs consist of aluminum chloride and [EMIm]Cl are more extensively studied for AIBs. Their ionic conductivity is approximately 10^−2^ S cm^−1^, comparable to or higher than the organic electrolytes currently used in LIBs.^25^ In this electrolyte, the aluminum plating process is affected by the molar ratio of AlCl_3_ to [EMIm]Cl and the balance of anion species. Aluminum exists in the form of aluminum complexes (carrier ions are AlCl_4_^−^ and Al_2_Cl_7_^−^), which act as monovalent ion counterparts rather than free Al^3+^. The reversible reaction of aluminum stripping and plating can be expressed as follows:^67^

1 4Al_2_Cl_7_^−^ + 3e^−^ ↔ Al + 7AlCl_4_^−^

It is worth noting that for the AlCl_3_/[EMIm]Cl IL electrolyte, the presence of carrier ion species in the electrolyte depends on the molar ratio of AlCl_3_ to [EMIm]Cl (denoted as *x*, defined as *x* = *n*(AlCl_3_)/*n*([EMIm]Cl)). Specific chemical reactions are as follows:^68^

2 [EMIm]Cl + *x*AlCl_3_ → [EMIm]^+^ + (1 − *x*)Cl^−^ + *x*AlCl_4_^−^ (*x* < 1)

3 [EMIm]Cl + *x*AlCl_3_ → [EMIm]^+^ + *x*AlCl_4_^−^ (*x* = 1)

**Fig. 3:**
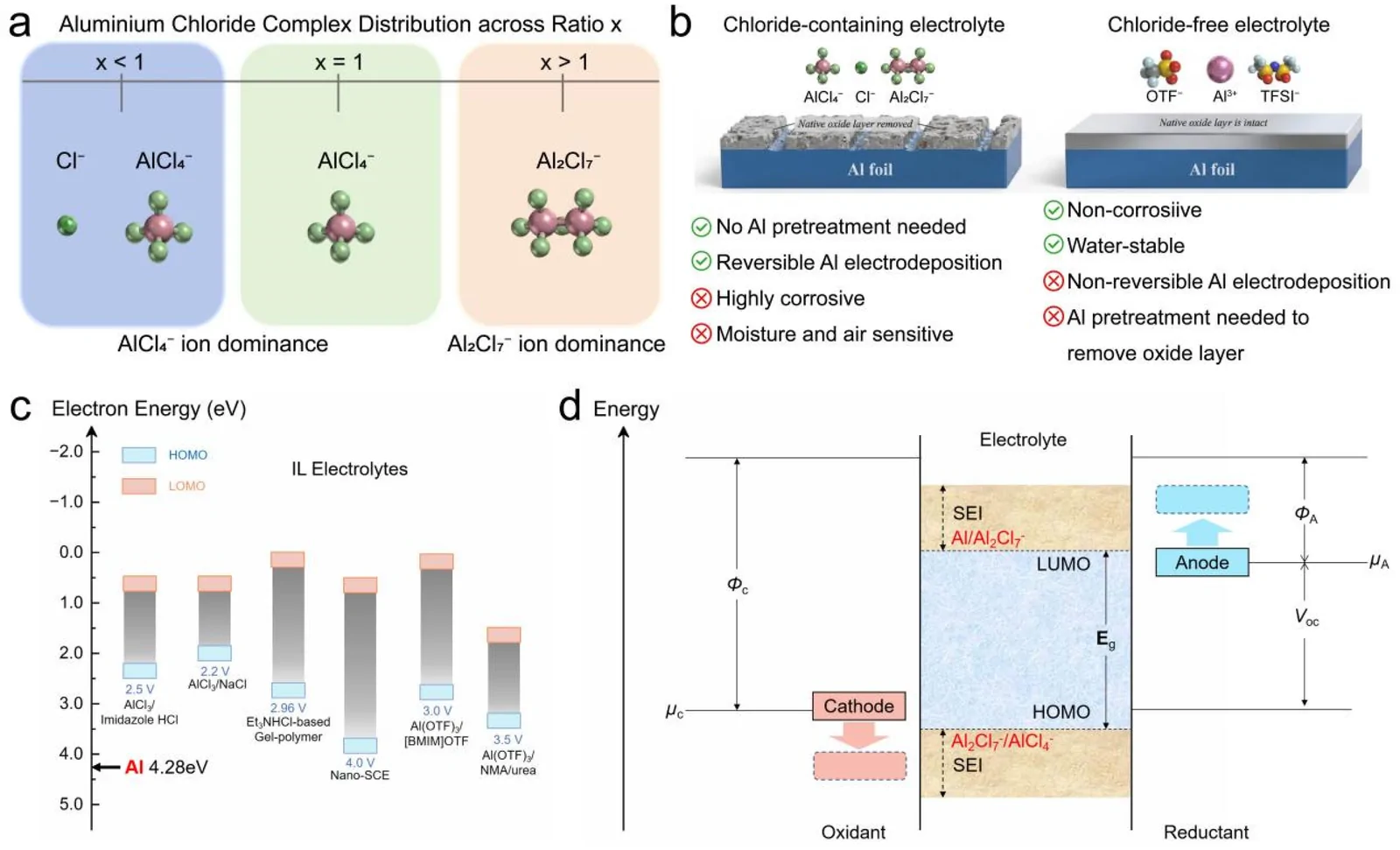
(a) Species distribution in AlCl_3_ : [EMIm]Cl at varying molar ratios (*x*). (b) Strengths and challenges linked to chloride-containing and chloride-free electrolytes. (c) Schematic summary of bandgaps and energy level positions for the Al anode and non-aqueous electrolytes. (d) Illustrated energy diagram at open-circuit for the AlCl_3_–[EMIm]Cl-based non-aqueous electrolyte.

4
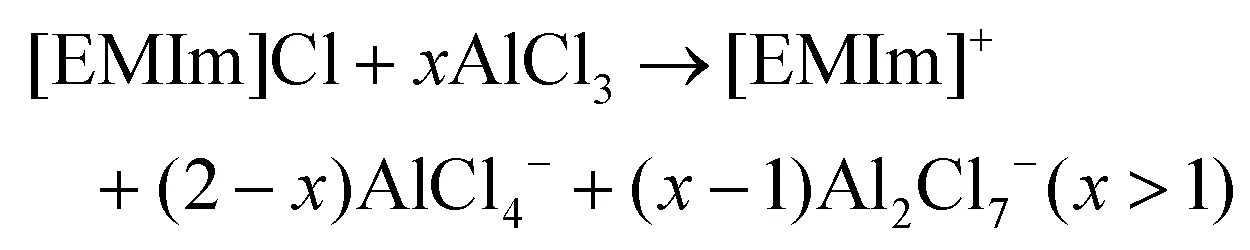
when *x* < 1, the electrolyte is basic, with AlCl_4_^−^ and Cl^−^ being the dominant species. When *x* = 1, the electrolyte is neutral, predominantly containing AlCl_4_^−^ (Fig. 3a). However, AlCl_4_^−^ not only has a more negative reduction potential than the organic cation, but the aluminum plating *via* AlCl_4_^−^ requires high temperatures to occur, which makes it impossible to utilize AlCl_4_^−^ to achieve reversible aluminum deposition/dissolution on the anode surface at room temperature (eqn (5)).^69–72^

5 AlCl_4_^−^ + 3e^−^ ↔ Al + 4Cl^−^

Consequently, Al_2_Cl_7_^−^ is considered the key active species for achieving the reversible aluminum plating and stripping process through the reaction described by eqn (1).^73^ Specifically, Al_2_Cl_7_^−^ not only possesses an asymmetric structure and exhibits high activity in the aluminum plating process but also eliminates the non-conductive oxide film on the aluminum surface while enhancing active nucleation sites (Fig. 3b).^74^ In summary, the capacity of AIBs using IL electrolytes primarily originates from the anolyte and depends mainly on the availability of redox-active species (especially Al_2_Cl_7_^−^). The aluminum metal electrode serves only as a substrate for the aluminum plating/stripping processes. To ensure the stable existence of Al_2_Cl_7_^−^ anions in the IL electrolyte, the ratio of aluminum chloride to [EMIm]Cl should be greater than 1 (*x* > 1, the electrolyte is acidic).

**Fig. 4:**
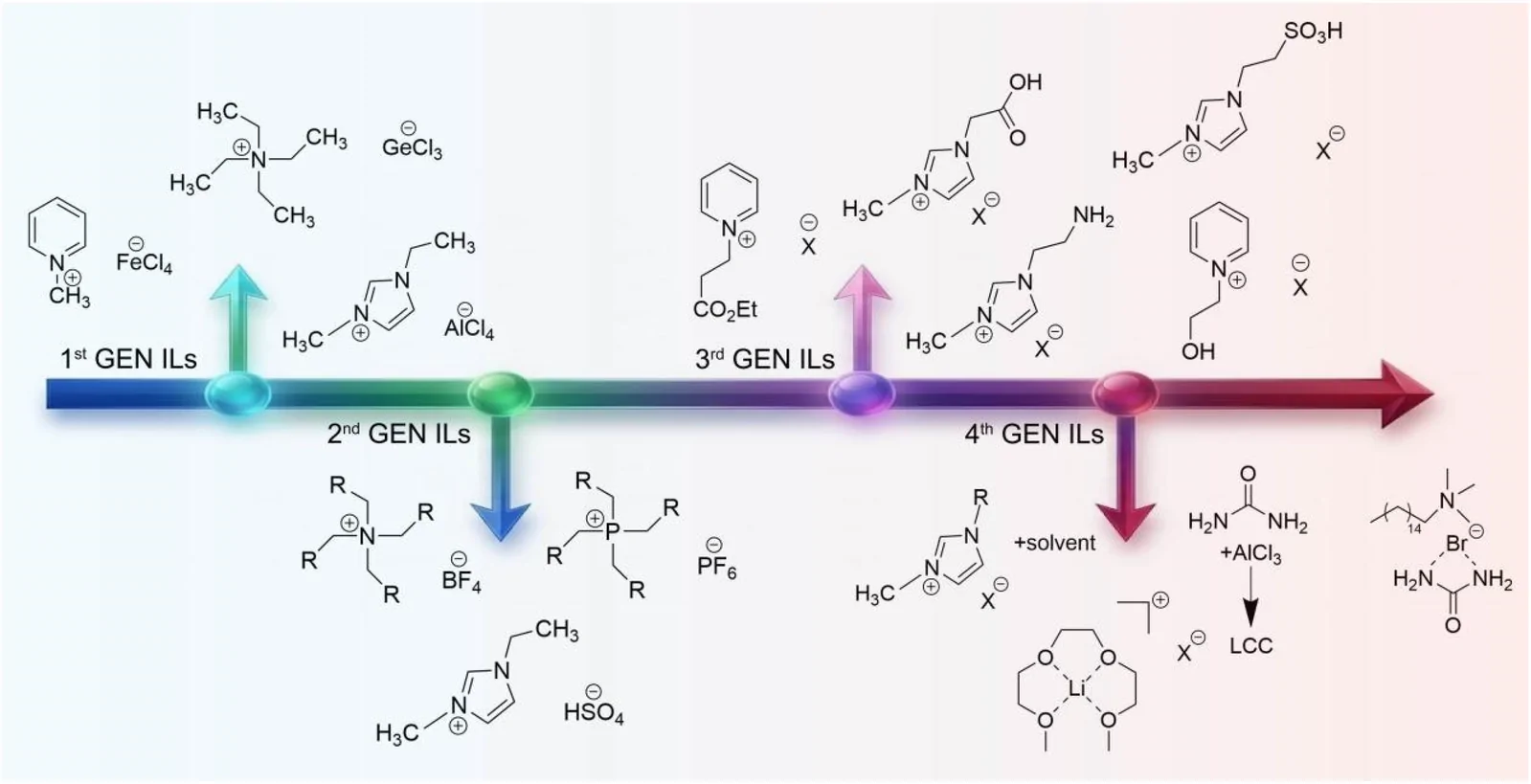
Development of ILs from the first to fourth generations with analogues appearing in the latest generation.

Notably, the earliest studied chloroaluminate-based ILs are generally categorized as the first generation of ILs due to their extreme sensitivity to air and humidity.^49^ Despite their long research history, their applications in electrochemistry have been limited, primarily because batteries are typically assembled in sealed environments, and the high sensitivity and strong corrosivity of chloroaluminate-based ILs have hindered their development.^75^ In the following decades, researchers developed a series of room-temperature stable, air- and moisture-resistant ILs (*i.e.*, the second generation ILs), which have been gradually applied in the study of AIBs. By the end of the 20^th^ century, ILs were regarded as designable solvents with millions of possible cation and anion combinations, enabling performance customization for specific application needs, which laid the foundation for the third generation ILs (Fig. 4) and provided solid technical support and broad application prospects for the further development of IL electrolytes.^76,77^

## 3. Design principle of IL electrolytes for aluminum-ion batteries

As the cornerstone of AIBs technology, the design logic for IL electrolytes must be intricately tailored to the unique chemical signatures of aluminum ions and specific application requirements. The overarching objective is to maximize the performance advantages of aluminum-based systems while ensuring superior safety and prolonged cyclability. Consequently, the following principles should be integrated into the rational design of IL electrolytes for AIBs.

A broad ESW is the paramount feature distinguishing ILs from conventional aqueous or organic electrolytes. It defines the maximum potential difference tolerable at the electrode/electrolyte interface, thereby determining the threshold for parasitic electrochemical reactions.^78,79^Fig. 3c illustrates the relationship between bandgap energies and band positions for aluminum metal anodes across various non-aqueous media. For AIBs, a wide electrochemical stability window (ESW, typically ≥2.5 V) effectively suppresses side reactions, thereby preventing the unproductive consumption of active materials. This expansion of the operational voltage range establishes the chemical foundation for elevating overall energy density.^80,81^

Beyond thermodynamic stability, the chemical compatibility between ILs and electrode materials dictates the reaction kinetics and long-term durability. An ideal IL system should circumvent uncontrollable side reactions with the Al anode, cathode, or reactive intermediates, while possessing the capability for preferential, directional decomposition to form a thin, dense, and protective interphase.^82–84^ This interphase typically evolves into a structurally robust solid electrolyte interphase (SEI) at the anode and a cathode electrolyte interphase (CEI) at the cathode. Functioning as selective molecular sieves, these interphases inhibit continuous electrolyte degradation while facilitating rapid Al-ion transport, significantly reducing self-discharge and bolstering CE. Therefore, this synergistic mechanism is central to sustaining cycling stability and serves as an important consideration for evaluating electrolyte design. To further elucidate this, Fig. 3d depicts the relative electronic energy distribution in non-aqueous AIBs, where *Φ*_A_ and *Φ*_C_ represent the energy levels of the Al anode and cathode. The energy gap (*E*_g_) between the highest occupied molecular orbital (HOMO) and the lowest unoccupied molecular orbital (LUMO) defines the ESW. To enable stable SEI/CEI formation, the energy levels must satisfy the boundary conditions: *μ*_A_ > LUMO and *μ*_C_ < HOMO. Adherence to these criteria ensures a protective interfacial architecture for efficient, reversible operation.^85^

Even with optimized thermodynamics, the ionic conductivity and viscosity remain decisive parameters for rate capability and internal resistance.^86,87^ Due to the high charge density of Al ions, strong coulombic interactions with anions (*e.g.*, AlCl_4_^−^, Al_2_Cl_7_^−^) often lead to reduced ionic dissociation and insufficient carrier concentration. Coupled with the inherent high viscosity of ILs, their room-temperature conductivity typically trails behind conventional organic counterparts.^88–90^ Since ionic mobility is inversely proportional to viscosity (Stokes’ law), design strategies must break the viscosity-conductivity trade-off through component modulation, such as incorporating low-viscosity eutectic components or functional additives, to achieve rapid interfacial ion migration and optimal wettability.

With expanding application scenarios, environmental tolerance has become a design priority. On one hand, for sub-zero or polar applications, molecular engineering (*e.g.*, introducing flexible segments or low-melting-point eutectics) is required to depress the freezing point and prevent conductivity collapse. On the other hand, enhancing air and moisture stability is crucial to simplifying assembly processes and reducing reliance on strictly inert environments.^80,91^ Such improvements significantly lower environmental control costs and enhance the chemical stability of electrolytes during storage and operation.

Finally, cost and safety serve as the bridge connecting laboratory research to industrial production. Although IL electrolytes possess intrinsic safety characteristics (such as non-flammability, low vapor pressure, and low biological toxicity), their exorbitant raw material costs (primarily originating from expensive organic ligands or high-purity aluminum salts) severely restrict large-scale promotion. Therefore, economic feasibility analysis must be incorporated into the design principles, aiming to find the optimal balance between intrinsic safety and economic cost by developing IL systems based on inexpensive organic salts, optimizing aluminum salt ratios to reduce the dosage of corrosive components, or utilizing eutectic effects to decrease reliance on high-cost components, thereby truly pushing AIB technology from theory to the market.

## 4. Optimization strategies for IL electrolytes

**Fig. 5:**
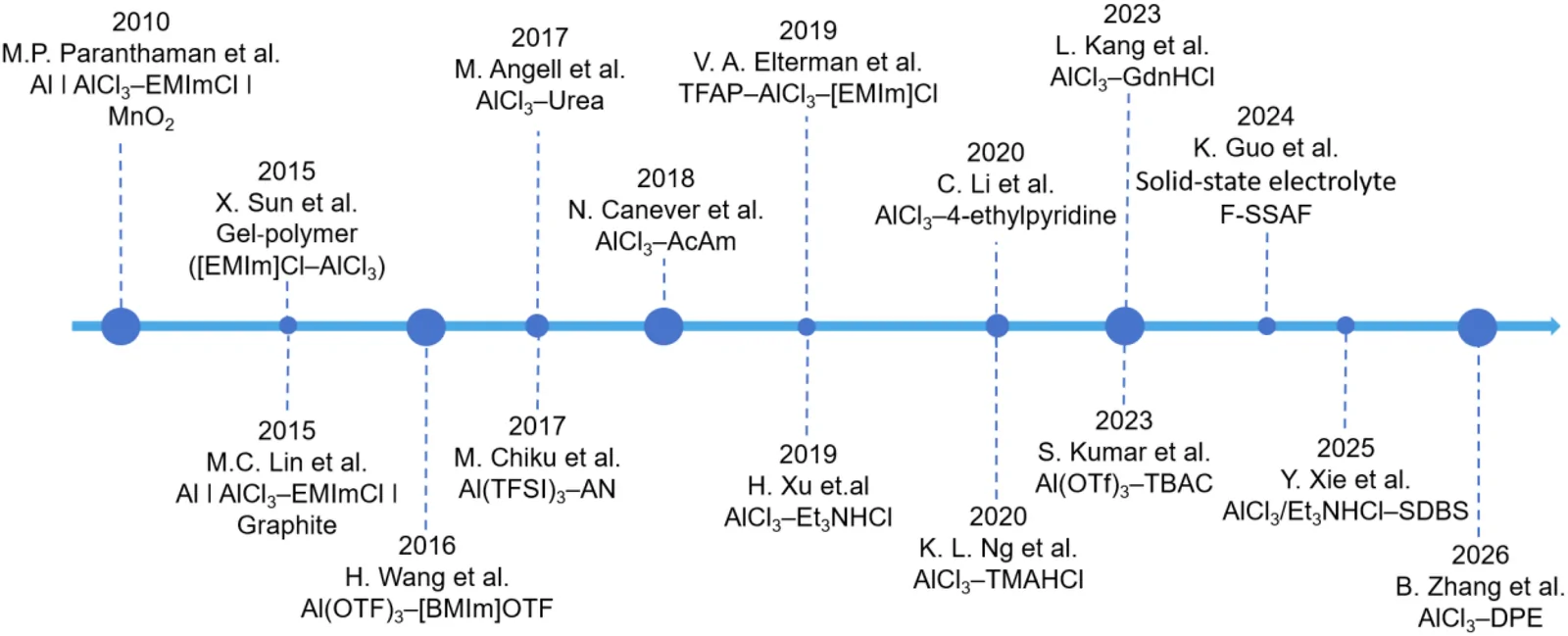
Timeline of ionic liquid electrolyte evolution for AIBs.

Despite the unique advantages of IL electrolytes in AIBs, their large-scale application still faces several intrinsic issues, such as high cost, excessive viscosity, strong corrosivity, and poor air stability. First, the high price of organic salt raw materials represented by imidazolium salts significantly increases the cost of the electrolyte, becoming a bottleneck for industrialization. Second, the high viscosity reduces the wettability of the electrolyte toward electrode materials, increases interfacial contact resistance, and limits the high-current charge–discharge performance of the battery. Furthermore, aluminum chloride-based ILs (such as AlCl_3_/[EMIm]Cl) exhibit strong corrosiveness, primarily originating from two aspects: on the one hand, active anions like Al_2_Cl_7_^−^ easily react with conventional metals such as aluminum, iron, nickel, titanium, and tungsten, with only a few noble metals like molybdenum, tantalum, and platinum remaining stable, which restricts the material selection for current collectors and battery casings. On the other hand, the strong Lewis acidity can corrode cathode materials containing transition metals or sulfur, leading to the dissolution of active substances and severely affecting cycling stability.^26,27,92^ Additionally, the hygroscopicity of anions triggers moisture-driven side reactions in the atmosphere, generating hydrochloric acid and explosive H_2_/chlorine gas mixtures through hydrolysis, potentially leading to battery venting or catastrophic failure risks.^67,93^ To address these issues, current research on the modification of IL electrolytes focuses on the following four aspects, which will be discussed in detail in subsequent sections: (1) selection of cation–anion pairs and basic formula design, (2) introduction of functional additives and interfacial regulation, (3) optimization of eutectic solvent systems, and (4) construction of gel and quasi-solid-state structures to promote solid-state transformation. A chronological overview of these evolving strategies, highlighting the progression from fundamental electrolyte formulations to advanced architectures, is illustrated in the timeline (Fig. 5). Table 1 complements this overview by summarizing the electrochemical performance metrics of representative IL electrolyte systems in both symmetric Al‖Al cells and full cells. This comparative data underscores that these strategies markedly enhance the reversible cycling of aluminum anodes while concurrently enabling the full realization of overall cell performance in AIBs. A detailed discussion of each optimization strategy follows.

**Table 1:** Comparison of the performance metrics of different electrolytes for rechargeable AIBs

Electrolyte	Symmetric cell		Full cell				Ref.
	Current density and capacity	Cycle time (h)	Cathode	Current density (A g^−1^)	Specific capacity (mAh g^−1^)	Cycle number	
AlCl_3_–[EMIm]Cl (1.3 : 1)	—	—	Graphitic-foam	4	∼70	∼7500	23
Al(OTF)_3_–[BMIm]OTF	—	—	V_2_O_5_ nanowire	0.01	40	∼20	94
AlCl_3_–[EMIm]Br	—	—	S@CMK-3	0.251	∼200	10	95
AlCl_3_–NBMPBr (1.3 : 1)	—	—			∼400	35	
AlCl_3_–[Et_3_NH]Cl (1.5 : 1)	—	—	Graphene aerogel	5	112	30 000	34
AlCl_3_–TMAHCl	—	—	GNP-1000	2	134	3000	96
AlCl_3_–[EMIm]Cl : [BMIm]Cl 2 : 1	—	—	Natural graphite	0.01 (−20 °C)	∼110	—	97
AlCl_3_–[EMIm]Cl (1.5 : 1) + 45% benzene (v/v)	—	—	Graphite	5	∼90	1000	98
AlCl_3_–[EMIm]Cl (1.3 : 1) + 2 wt% NaCl	0.054 mA cm^−2^	600	S/graphene	0.1	473	50	99
	0.27 mAh cm^−2^						
AlCl_3_–[Et_3_NH]Cl (1.5 : 1) + 1 wt% CTAC	3 mA cm^−2^	∼1200	Flake graphite	0.5	105	600	100
	1 mAh cm^−2^						
AlCl_3_–[EMIm]Cl–dFBn (1.3 : 1 : 0.8)	0.054 mA cm^−2^	1000	Graphene@S-34%	0.1	507	300	101
	0.27 mAh cm^−2^						
AlCl_3_–[Et_3_NH]Cl (1.5 : 1) + 0.5 wt% SDBS	1 mA cm^−2^	1000	Flake graphite	0.5	90.01	1000	102
	5 mAh cm^−2^						
AlCl_3_–[EMIm]Cl (1.3 : 1) + 45% dichloromethane (v/v)	—	—	Graphite	0.1	127.9	1600	88
AlCl_3_–[EMIm]Cl–FB (1.3 : 1 : 5)	8 mA cm^−2^	1500	MoSe_2_	0.1	∼60	∼50	103
	8 mAh cm^−2^						
AlCl_3_–urea (1.3 : 1)	—	—	Graphite	0.1	73	180	104
AlCl_3_–*N*-ethyl urea (1.4 : 1)	—	—	Graphite	0.1	∼76	∼80	105
AlCl_3_–4-ethylpyridine (1.3 : 1)	—	—	Natural graphite	0.025	∼80.75	1000	106
AlCl_3_–[GdnH]Cl (1.8 : 1)	—	—	Natural graphite	1	∼109	1000	107
AlCl_3_–AcA (1.3 : 1)	—	—	S@CMK-3	0.1	500	60	108
AlCl_3_–AcA–dFAcA (1.3 : 1 : 0.03)	0.1 mA cm^−2^	∼225	S@mesoporous graphene	1	255.1	300	109
	0.1 mAh cm^−2^						
4 mol kg^−1^ AlCl_3_ in dipropyl ether	0.2 mA cm^−2^	2000	Mo_6_S_8_	0.05	∼70	300	110
	0.1 mAh cm^−2^						
AlCl_3_–[EMIm]Cl (1.3 : 1) − 35 wt% MOF	0.05 mA cm^−2^	∼200	Graphite	0.1	62	2000	111
AlCl_3_–[EMIm]Cl (1.5 : 1) + FEC + AlF_3_ SPE	0.1 mA cm^−2^	4000	Graphite	0.05	∼80	300	112
	0.1 mAh cm^−2^						
AlCl_3_–[Et_3_NH]Cl (1.6 : 1) − 20 wt% PAM GPE	—	—	Flake graphite	2 (charge)/1 (discharge)	89	800	113
AlCl_3_–[EMIm]Cl (1.7 : 1) − 4.8 wt% PA GPE	0.5 mA cm^−2^	800	Graphite	0.5	94	4200	114
	0.5 mAh cm^−2^						
AlCl_3_–[EMIm]Cl (1.5 : 1) − 10 wt% AAM cross-linked GPE	—	—	3D graphene	10	122	20 000	115
AlCl_3_–[EMIm]Cl (1.3 : 1) − NIPA − DMAA GPE	0.5 mA cm^−2^	8000	Graphite	2	∼60	50 000	116
	0.5 mAh cm^−2^						
AlCl_3_–Guan–Cl (1.8 : 1) − 20 wt% PA GPE	0.1 mA cm^−2^	450	Sulfur with natural graphite	0.05	1000	25	63

### 4.1. Cation–anion pair engineering for fundamental formulation

A key innovation in the field of IL electrolyte development is the replacement of metal cations (such as Na^+^ or Ca^2+^) in molten salts with long-chain organic cations.^70,117^ This substitution lowers the melting point, enabling IL electrolytes to operate stably at room temperature. The types and coordination modes of cations and anions in ILs have a significant impact on their chemical/physical properties (such as viscosity, melting point, conductivity, hydrophilicity, hydrophobicity, *etc*.).^118–120^ Imidazolium cations (Im^+^) with different alkyl chains, such as 1-butyl-3-methylimidazolium (BMIm^+^) and 1-ethyl-3-methylimidazolium (EMIm^+^), are commonly used electrolyte cations in AIBs.^121–123^ Since chloride ions (Cl^−^) possess superior conductivity and stability compared to other anions, current IL electrolytes predominantly employ chloride-based systems. Although AlCl_3_-[EMIm]Cl is most widely applied in AIBs, it still suffers from issues such as high cost, strong corrosivity, poor low-temperature performance, and sluggish kinetic response. To overcome these drawbacks, researchers attempt to optimize performance by adjusting the composition of ILs.

**Fig. 6:**
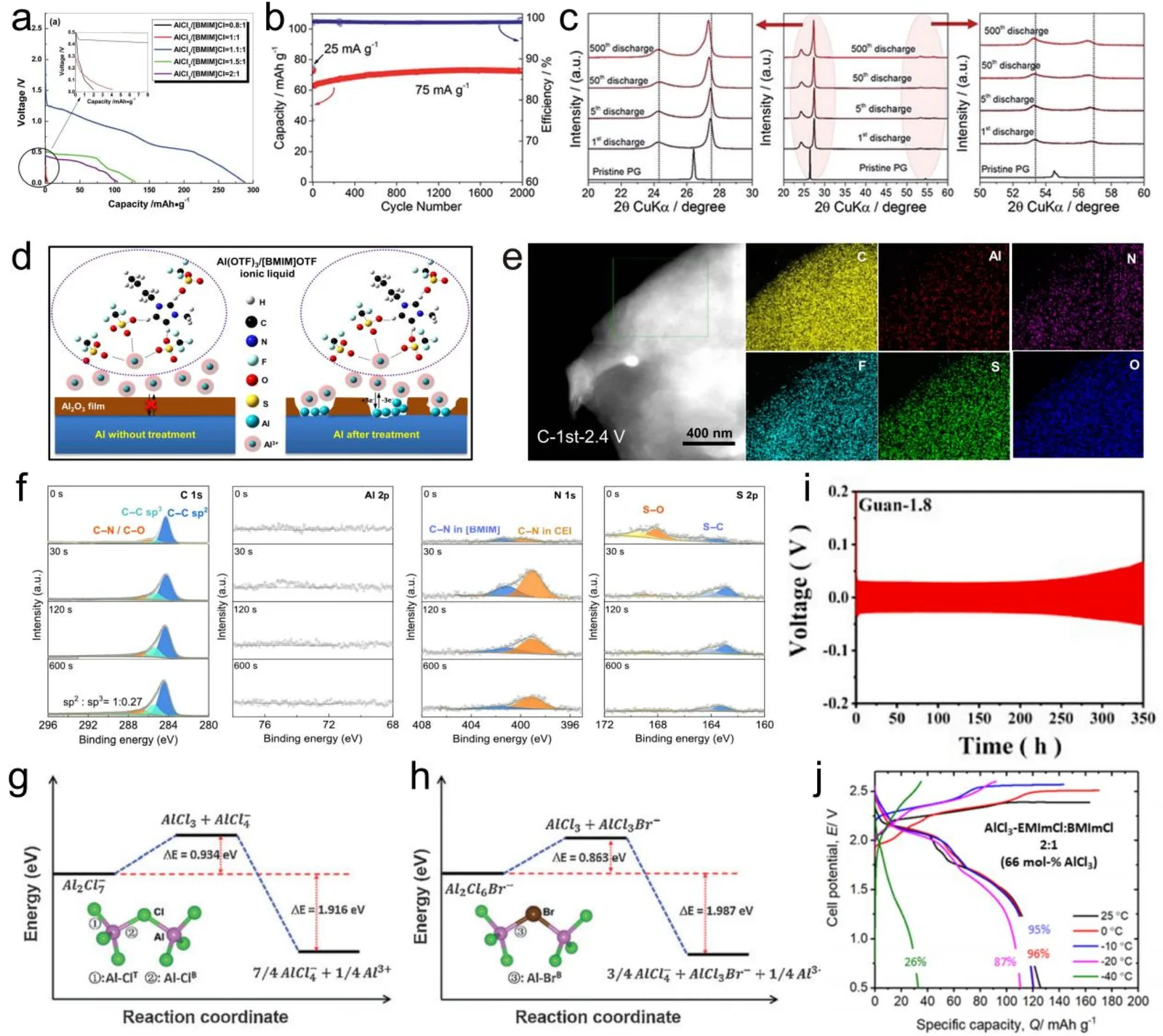
(a) Initial discharge curves of AIBs in AlCl_3_/[BMIM]Cl ILs at varying molar ratios. Reproduced with permission.^124^ Copyright 2015, The Royal Society of Chemistry. (b) Cycling performance of the Al/EMIMCl : AlCl_3_/PG full cell at 25 mA g^−1^. (c) Ex situ XRD patterns of PG electrodes after varying cycles. Reproduced with permission.^125^ Copyright 2017, The Royal Society of Chemistry. (d) Schematic of Al deposition/dissolution processes on different Al anode surfaces. Reproduced with permission.^94^ Copyright 2016, American Chemical Society. (e) The TEM image, elemental map, EDX spectra and (f) XPS depth profiles of the rGO cathode a in the charge stage at 2.4 V. Reproduced under terms of the CC-BY license.^126^ Copyright 2024, Tsinghua University Press. Energy profiles of the dissociation processes for (g) Al_2_Cl_7_^−^ and (h) Al_2_Cl_6_Br^−^ anions. Reproduced with permission.^95^ Copyright 2018, Wiley-VCH. (i) Galvanostatic charge/discharge cycling of symmetric cells using Guan-1.8 electrolyte. Reproduced with permission.^63^ Copyright 2026, Elsevier. (j) Galvanostatic charge/discharge profiles of full cells tested at 10 mA g^−1^ across temperatures from 25 to −40 °C. Reproduced with permission.^97^ Copyright 2023, American Chemical Society.

The type and ratio of anions in ILs exert a decisive influence on the electrochemical performance of rechargeable AIBs. To systematically analyze the anion effect, Wang *et al.*^124^ conducted an in-depth investigation into the structure–property relationships of chloroaluminate-based ILs by modulating the molar ratio of AlCl_3_ to imidazolium chloride. Research results indicate that the ESW of the electrolyte narrows as the reducing power of halide ions increases, a trend further validated by density functional theory (DFT) calculations. Simultaneously, the coexistence of multiple chloroaluminate anions, such as Cl^−^, AlCl_4_^−^, and Al_2_Cl_7_^−^, is universally observed in ILs with different formulations. In a battery system employing V_2_O_5_ nanowires as the cathode, the electrolyte exhibits optimal performance when the AlCl_3_/[BMIM]Cl molar ratio is optimized to 1.1 : 1, with the initial discharge plateau stabilizing at approximately 1.0 V and a discharge capacity reaching 288 mAh g^−1^. The study explicitly points out that the concentration of Al_2_Cl_7_^−^ is key to regulating its physicochemical properties and electrochemical behavior (Fig. 6a). Subsequently, in 2017, Hahn's group^125^ employed an acidic IL electrolyte with an AlCl_3_/[BMIM]Cl molar ratio of 1.5 : 1 in an aluminum–graphite dual-ion battery system. This high AlCl_3_ ratio system, with Al_2_Cl_7_^−^ as the primary active anion, can drive rapid and reversible intercalation/deintercalation reactions within graphite layers (Fig. 6c). At a current density of 25 mA g^−1^, the battery exhibits an initial charge–discharge efficiency of approximately 70%, with the CE rapidly climbing to nearly 96% after several activation cycles, maintaining an average operating voltage of about 1.9 V. Benefiting from the high diffusion coefficient and structural stability of Al_2_Cl_7_^−^, the system operates stably for over 2000 cycles at a high rate of 75 mA g^−1^ (exceeding five months of testing), with the capacity showing a gradual upward trend during cycling, demonstrating excellent rate performance and ultra-long cycle life (Fig. 6b). This study fully proves that increasing the AlCl_3_ proportion in acidic ILs can significantly optimize the reaction kinetics of anion-intercalation cathodes, providing key electrolyte design evidence for developing AIBs with both high power output and long cycling stability.

The strong corrosivity of ILs primarily originates from highly active chloroaluminate anions such as Al_2_Cl_7_^−^. Therefore, employing non-chloride components is the core strategy to reduce corrosion risk. As early as 2016, Wang *et al.*^94^ proposed a chloride-free and water-stable IL electrolyte based on aluminum trifluoromethanesulfonate (Al(OTF)_3_) and 1-butyl-3-methylimidazolium trifluoromethanesulfonate ([BMIm]OTF). This electrolyte exhibits a high oxidation potential of 3.25 V (*vs.* Al^3+^/Al), combining high ionic conductivity with excellent electrochemical stability (Fig. 6d). Recently, Zheng *et al.*^126^ developed a non-corrosive system based on 0.5 mol L^−1^ Al(OTF)_3_/[BMIm]OTF, paired with a three-dimensional porous reduced graphene oxide (rGO) cathode and an aluminum anode. This electrolyte is free of chloroaluminate ions, fundamentally avoiding the corrosion of battery components by Al_2_Cl_7_^−^, while possessing good water stability and a wide electrochemical window. Experiments show that the Al‖rGO-150 battery can operate stably for 2700 cycles at 100 mA g^−1^, and the charge-transfer resistance gradually decreases with cycling, reflecting a stable interfacial structure. Mechanism studies indicate that Al^3+^ based species can reversibly intercalate/deintercalate between rGO layers, while Al(OTF)_*x*_ (*x* < 3) and [BMIm]^+^ contribute to the capacity through adsorption at oxygen-containing defect sites. Quasi-*in situ* Raman, X-ray photoelectron spectroscopy (XPS), and transmission electron microscopy (TEM) analyses collectively confirmed that the Al intercalation/deintercalation process is accompanied by local disorder changes in rGO, while the host skeleton remains intact. The CEI layer, primarily composed of decomposition products from [BMIm]^+^ and OTF^−^, uniformly covers the cathode surface (Fig. 6e and f). This system achieves aluminum deposition/dissolution without chloride pretreatment, highlighting the application prospects of chloride-free, non-corrosive liquid electrolytes in high-safety aluminum batteries. To further weaken corrosivity, bromide ions can partially replace chloride ions, as the dissociation energy of Al_2_Cl_6_Br^−^ is lower than that of Al_2_Cl_7_^−^ (reduced by about 15 times), which helps accelerate reaction kinetics (Fig. 6g and h).^95^ Aluminum-sulfur batteries using modified electrolytes exhibit an initial discharge capacity as high as 1500 mAh g^−1^. Furthermore, aluminum bis(trifluoromethylsulfonyl)imide (Al(TFSI)_3_) can also achieve a wide electrochemical window of 3.6 V in acetonitrile solvent, which is superior to traditional chloride systems and significantly promotes the uniform deposition/dissolution of aluminum.^127^ Although these non-corrosive systems significantly improve the hygroscopicity and electrochemical performance of IL electrolytes, most of them still require anode surface pretreatment with chloride precursors due to their inefficiency in opening ion channels through the alumina passivation film. This increases the cost and operational difficulty of battery construction.^128,129^

To reduce the cost of IL electrolytes and expand their operating temperature range, researchers are continuously exploring new IL systems. In terms of cost control, organic salts such as triethylamine hydrochloride (Et_3_NHCl) and trimethylamine hydrochloride (TMAHCl) have become low-cost replacement candidates for imidazolium salts, as their prices are only 1/20 to 1/30 of that of [EMIm]Cl.^34,96^ The IL analogues formed by compounding these salts with AlCl_3_ not only possess significant cost advantages but also exhibit higher oxidative decomposition potentials than the traditional AlCl_3_/[EMIm]Cl system. Recently, Tsai *et al.*^63^ constructed IL analogous electrolytes by directly mixing aluminum trichloride and guanidine hydrochloride (Guan-Cl) at different molar ratios (1 : 1.6, 1 : 1.8, 1 : 2.0). These were applied in aluminum–graphite full-cell systems to achieve optimization of cost and electrochemical performance. While this approach significantly reduces the reliance on expensive imidazolium salts, aligning with the Green Chemistry principle of *Less Hazardous Chemical Syntheses*, the residual corrosivity and chloride content still require careful lifecycle management. Nevertheless, the substantially lower material cost and enhanced electrolyte stability represent an important step toward more sustainable and economically viable AIBs. In the constructed aluminum–graphite batteries, the Guan-1.8 electrolyte shows excellent cycling stability, operating continuously for 125 cycles without short-circuiting, while achieving a CE as high as 99.8%. Furthermore, symmetric cell tests revealed that Guan-1.8 maintains a stable polarization voltage and operates steadily for 350 h at a current density of 0.1 mA cm^−2^ (Fig. 6i). Regarding environmental adaptability, Schoetz *et al.*^97^ designed a mixed imidazolium cation electrolyte suitable for low temperatures down to −40 °C. By leveraging the synergistic effect between [EMIm]^+^ and [BMIm]^+^, the symmetry of the single-cation system was broken, reducing the viscosity of the electrolyte at −40 °C from 925 cP to 332 cP. Based on this, the aluminum–graphite battery employing AlCl_3_-[EMIm]Cl : [BMIm]Cl (66 mol% AlCl_3_) maintained 87% and 26% of its capacity at −20 °C and −40 °C, respectively (Fig. 6j), performance significantly superior to that of the single [EMIm]Cl based system. Imidazolium salts are generally non-biodegradable, and their synthesis often involves energy-intensive processes. However, their improved low-temperature performance can reduce the energy required to maintain battery operation under cold conditions, offering a potential use-phase energy benefit.

Despite the effectiveness of cation–anion engineering in tailoring electrolyte properties, the inherent corrosivity of chloroaluminates and the passivation issues of chloride-free systems continue to challenge interface stability. Consequently, regulating the electrode–electrolyte interface through functional additives has emerged as a complementary strategy to mitigate these interfacial limitations.

### 4.2. Functional additive incorporation for performance enhancement

In the research of rechargeable AIBs, the introduction of functional additives has been widely recognized as an effective approach to overcoming the limitations of IL electrolytes. Despite their high electrochemical stability and wide temperature adaptability, several bottlenecks remain: high viscosity limits ion transport, strong corrosivity leads to the degradation of electrodes and current collectors, and sensitivity to air and moisture reduces overall stability.^130–132^ To address these issues, researchers have developed various functional additives, whose optimization strategies for the aluminum anode and interfacial performance can be summarized into three categories. The first category aims to improve ion transport and deposition behavior. By introducing low-viscosity organic solvents to dilute the system, ion-pair interactions are reduced, thereby enhancing conductivity and deposition uniformity while maintaining the AlCl_4_^−^/Al_2_Cl_7_^−^ equilibrium, which optimizes rate performance and deposition morphology. The second category focuses on regulating the SEI structure to suppress side reactions. Additives can construct a stable protective layer on the Al anode surface, preventing the accumulation of by-products, mitigating corrosion, and reducing irreversible capacity loss, thus significantly enhancing cyclic CE and lifespan. The third category regulates deposition kinetics through surface adsorption. Certain additives (such as surfactants and organic molecules with specific functional groups) can form a dynamic adsorption layer on the electrode surface. Through steric hindrance and electrostatic effects, they inhibit electrolyte's corrosion to aluminum while modulating the interfacial electric field and ion transport kinetics. This balances nucleation and deposition rates, avoids local ion enrichment or depletion, induces uniform planar deposition of aluminum, and effectively suppresses dendrite growth. Notably, through these three strategies, functional additives strengthen the interaction between the IL and the aluminum anode from the dimensions of ion transport and interfacial protection, providing a feasible path for realizing high-stability and long-life AIBs.

Taking the typical AlCl_3_/[EMIm]Cl = 1.5 ratio as an example, the viscosity of this system at 20 °C is approximately 19.4 mPa s, which is significantly higher than the common range of current commercial LIB electrolytes (generally <10 mPa s).^101^ The intrinsic high viscosity of IL electrolytes significantly limits the migration and diffusion rates of ions in the electrolyte, which in turn affects the chemical reactions at the electrode/electrolyte interface.^133,134^ To leverage the advantages of high charge/discharge rates and high capacity in rechargeable AIBs, ensuring rapid ion diffusion and charge-transfer kinetics is an essential requirement. Therefore, reducing viscosity and enhancing transport properties through electrolyte engineering has become a key approach to overcoming the application bottlenecks of this system. At the mechanistic level, organic molecules primarily act as molecular diluents in IL electrolytes. Diluents have been extensively studied in organic electrolyte systems such as LIBs. Their main role is to weaken the strong electrostatic coupling of ion pairs or complex ions through molecular-level intercalation and interaction, effectively lowering system viscosity and improving ionic conductivity and transport kinetics while preserving key ionic species and reactivity, thereby improving battery rate performance, reducing polarization, and extending cycle life.^135–138^ In the AlCl_3_/[EMIm]Cl system, these molecules can enter the space between Al_2_Cl_7_^−^ anions and [EMIm]^+^ cations. Through hydrogen bonding and dipole–dipole interactions, they increase the spacing between ion pairs, thereby weakening coulombic attraction and ion aggregation effects.^139^ This structural relaxation not only directly reduces the macroscopic viscosity of the system but also significantly increases the proportion and mobility of free ions, leading to improved ionic conductivity. Meanwhile, the low-viscosity environment can reduce the concentration polarization of ions at the electrode/electrolyte interface and lower the interfacial impedance, thereby improving the power output under high-rate charge/discharge conditions, which is beneficial for the development and application of AIBs.^62,69,140^ Current research reports have confirmed that several organic molecules are successfully compatible with the AlCl_3_/[EMIm]Cl system and can significantly improve the ion diffusion coefficient, conductivity, and interfacial charge transfer kinetics.^98,101,103,141^ Notably, certain molecules containing halogen or aromatic ring structures can not only significantly reduce the electrolyte's viscosity but also stabilize the equilibrium between AlCl_4_^−^ and Al_2_Cl_7_^−^ to some extent due to their weak coordination with chloroaluminate ions. This maintains the thermodynamic stability of the system while enhancing ionic kinetics.

**Fig. 7:**
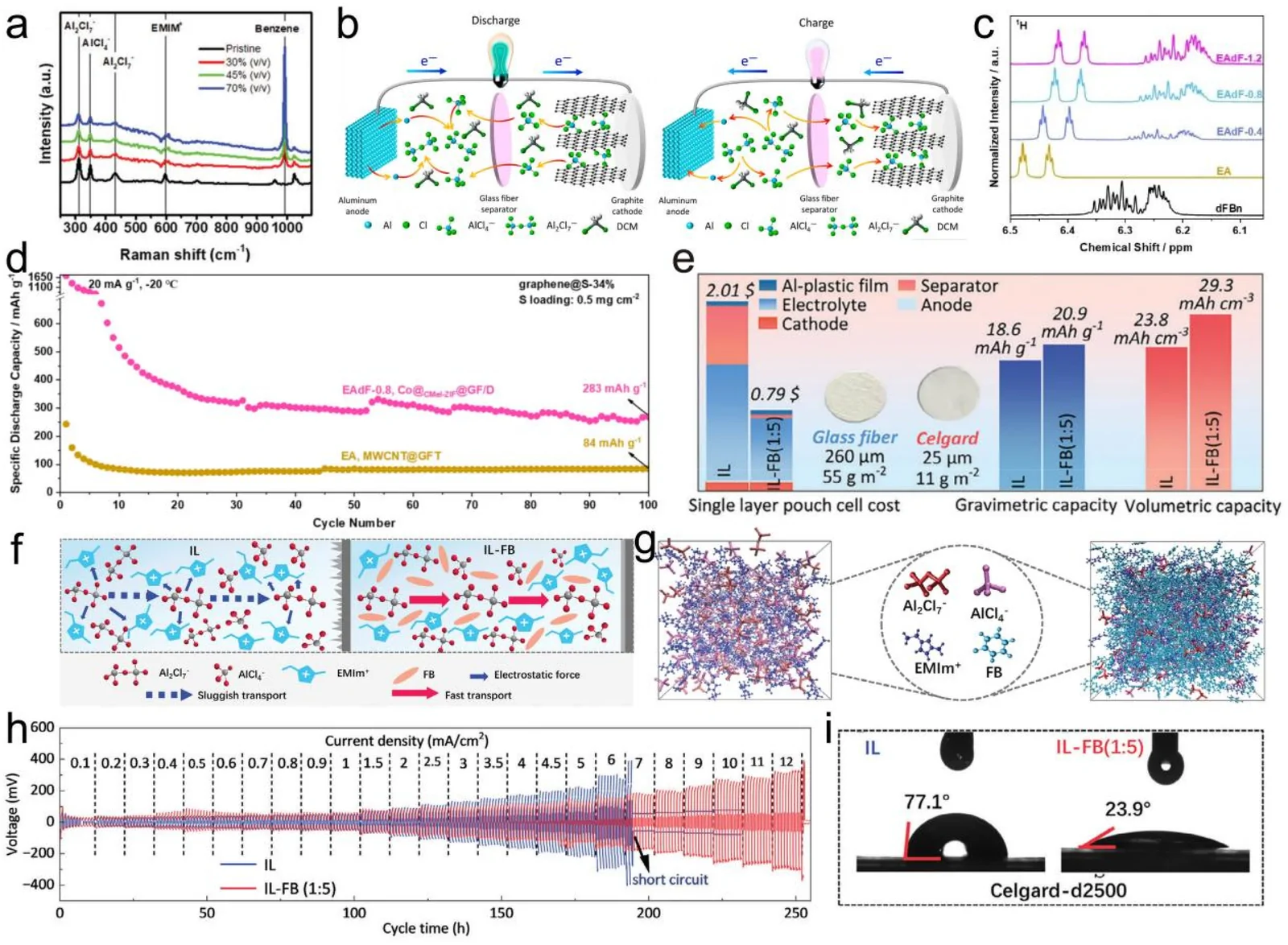
(a) FT-Raman spectra of the electrolyte with varying benzene addition ratios (v/v). Reproduced with permission.^98^ Copyright 2020, Royal Society of Chemistry. (b) Schematic of the charge/discharge process in the cell using AlCl_3_/[EMIm]Cl (molar ratio: 1.3 : 1) with DCM additive. Reproduced with permission.^88^ Copyright 2025, Elsevier. (c) ^1^H NMR spectra of the electrolytes. (d) Discharge specific capacity during cycling at the current density of 20 mA g^−1^ at −20 °C. Reproduced with permission.^101^ Copyright 2024, Wiley-VCH. (e) The volumetric capacity comparison, gravimetric capacity, and cost of the single-layer pouch cells using IL or IL-FB (1 : 5). (f) Simulation and analysis of diffusion kinetics and solvent structures for IL and IL-FB. (g) MD snapshots of IL and IL-FB. (h) Galvanostatic charge–discharge profiles of symmetrical cells at current densities for different electrolytes. (i) Contact angle test of different electrolytes with d2500. Reproduced with permission.^103^ Copyright 2025, Wiley-VCH.

As early as 2020, Tak and his collaborators^98^ used the AlCl_3_/[EMIm]Cl IL as a base, employed various organic solvents as diluents for the IL electrolyte, and systematically explored their impact on battery performance (Fig. 7a). Research results show that benzene molecules can reduce viscosity by weakening the interaction strength of ion pairs between Al_2_Cl_7_^−^ and [EMIm]^+^, increasing the ionic conductivity of the electrolyte to 2.43 S m^−1^. The hybrid electrolyte constructed by introducing benzene (45% v/v) as a diluent can achieve a specific capacity of 90 mAh g^−1^ at a high rate of 5 A g^−1^ and operate stably for over 1000 cycles. Benzene possesses electrochemical and chemical stability in ILs, optimizing transport solely through physical regulation, providing a paradigm for the viscosity engineering of IL electrolytes. In 2023, Kosar *et al.*^141^ utilized molecular dynamics (MD) simulations to study the effects of adding dichloromethane (DCM) or toluene to AlCl_3_/[EMIm]Cl IL electrolytes under acidic, neutral, and basic conditions. Data obtained from diffusion calculations indicate that the diffusion coefficient of Al^3+^ reaches 4.0 × 10^−12^ m^2^ s^−1^ when DCM is added to the acidic system. Detailed calculations show that DCM can effectively promote the formation of AlCl_4_^−^/Al_2_Cl_7_^−^, particularly in acidic mixtures, which in turn enhances the conductivity of the electrolyte system. In 2025, Wang's group^88^ experimentally verified the significant effect of DCM in reducing the viscosity of IL electrolytes. After adding 45% v/v DCM, the initial discharge capacity of the battery at 100 mA g^−1^ increased from 81.0 mAh g^−1^ without DCM to 131.5 mAh g^−1^, and only decayed to 127.9 mAh g^−1^ after 1600 cycles, while the CE remained near 100%. The performance improvement is attributed to the fact that the addition of DCM effectively promoted ion transport and interfacial charge transfer (Fig. 7b). The system viscosity decreased from 10.42 cP to 4.37 cP, the ionic conductivity rose from 21.34 mS cm^−1^ to 30.01 mS cm^−1^, and the charge-transfer resistance dropped from 99.2 Ω to 65.7 Ω. Furthermore, the addition of DCM improved the electrolyte wettability, thereby significantly enhancing the reversible intercalation/deintercalation kinetics of chloroaluminate anions in the graphite cathode.

Moreover, Xu *et al.*^101^ developed a localized high-concentration IL electrolyte (LCILE) by employing non-solvating 1,2-difluorobenzene (dFBn) as a co-solvent to dilute the AlCl_3_/[EMIm]Cl system (Fig. 7c). Experimental evidence indicates that the incorporation of dFBn substantially enhances the fluidity and ionic transport of the electrolyte. By facilitating electrostatic decoupling between ionic species while preserving the AlCl_4_^−^/Al_2_Cl_7_^−^ stoichiometric equilibrium, this approach ensures highly reversible Al plating/stripping. Consequently, the LCILE configuration empowers the battery with superior cycling durability, elevated specific capacity, and significantly attenuated electrochemical polarization. Therefore, the electrolyte exhibits superior physicochemical properties: the viscosity decreases from 18.5 mPa s (pure IL electrolyte) to 4.9 mPa s (EAdF-1.2), and the ionic conductivity reaches as high as 20.2 mS cm^−1^ at 20 °C (EAdF-0.8). In terms of electrochemical performance, the Al–S battery assembled with LCILE displays a high capacity of 507 mAh g^−1^ after 300 cycles at 20 °C. When combined with the Co@Cmel-ZIF@GF/D modified separator, the capacity remains at 283 mAh g^−1^ after 100 cycles at −20 °C (Fig. 7d). Similarly, Wu and his collaborators^103^ recently introduced the co-solvent fluorobenzene into the traditional AlCl_3_/[EMIm]Cl IL (optimal ratio 1 : 5) to form a hybrid electrolyte. The introduction of fluorobenzene (FB) helps weaken the electrostatic interactions between ions and promotes effective ion diffusion (Fig. 7f and g). The optimized hybrid electrolyte IL-FB (1 : 5) significantly increased the limiting current density (*i*_L_) from 7 mA cm^−2^ to 12 mA cm^−2^ (Fig. 7h), which ensures dense aluminum nucleus formation, uniform aluminum electrodeposition, and a highly reversible plating/stripping process. The assembled symmetric battery can cycle stably for 7500 hours at 8 mA cm^−2^ and 8 mAh cm^−2^. The Al‖Cu half cell maintained a CE of 99.5% after 2000 cycles at 5 mA cm^−2^. In addition, IL-FB significantly improved the wettability of the polypropylene (PP) separator, with the contact angle dropping from 77.1° to 23.9° (Fig. 7i), achieving compatibility between AIBs and commercial PP separators (Celgard 2500) for the first time, which can reduce the overall battery cost by 60% (Fig. 7e).

**Fig. 8:**
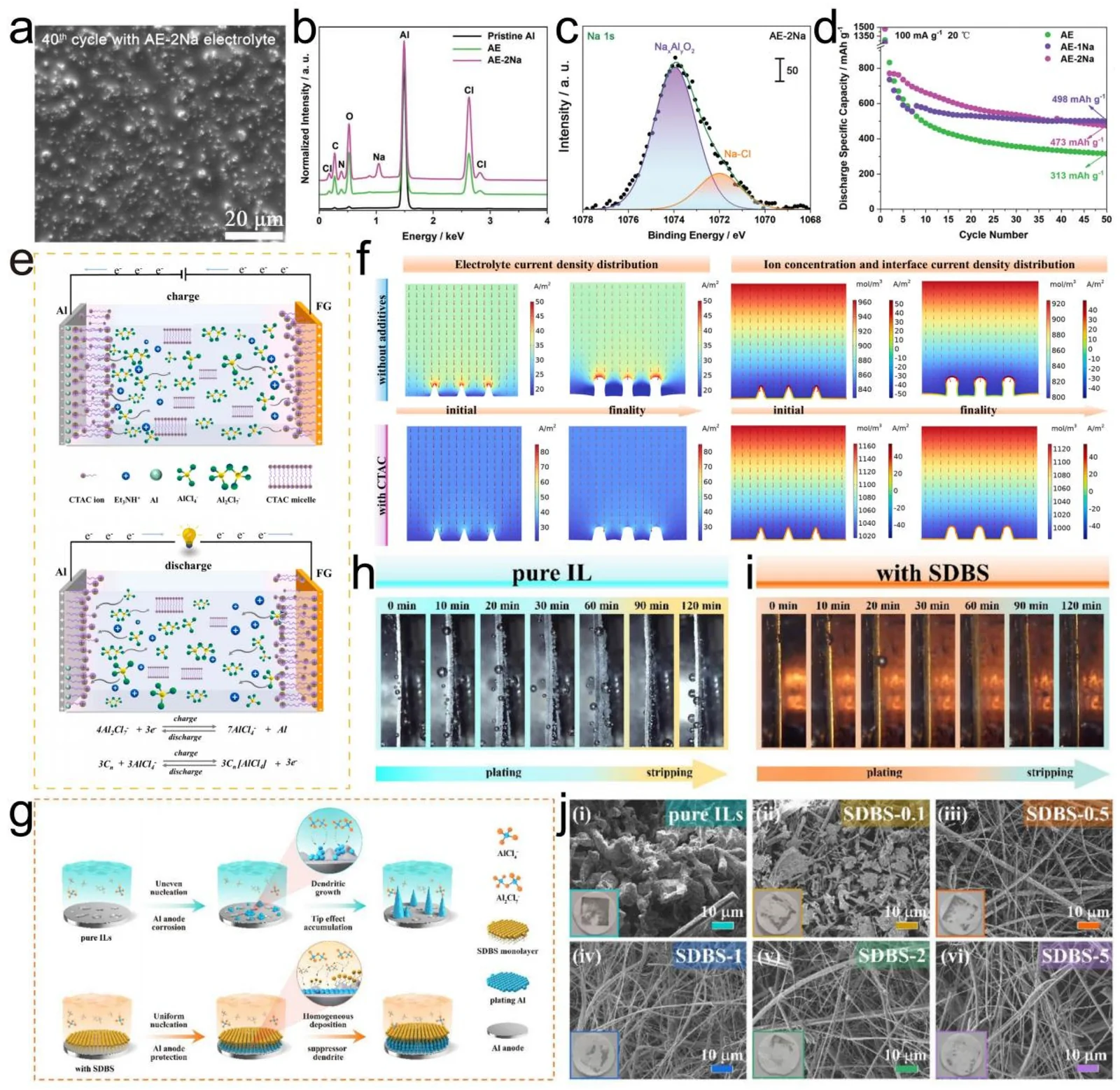
(a) SEM images of the Al electrode after 40 cycles with AE-2Na. (b) EDX spectra of the cycled Al electrode. (c) Na 1s XPS spectra of the Al anode after 40 cycles using AE-2Na as the electrolyte. (d) Discharge specific capacity of Al–S batteries with various electrolytes at 100 mA g^−1^. Reproduced with permission.^99^ Copyright 2023, Wiley-VCH. (e) Illustrated mechanism for constructing a molecular dynamic interface. (f) Distribution of current density and concentration in the electrolyte with/without CTAC additives. Reproduced with permission.^100^ Copyright 2024, Elsevier. (g) Schematic diagram of the Al deposition mechanism in different electrolyte systems. Optical microscopy observation of Al plating and stripping in (h) pure ILs and (i) SDBS-modified electrolyte. (j) SEM images of glass fiber separators from disassembled symmetric cells with different electrolytes after 100 cycles. Reproduced with permission.^102^ Copyright 2025, Wiley-VCH.

It is noteworthy that due to the complex decomposition behavior of IL electrolytes at the electrode/electrolyte interface and the significant uncertainty in their compatibility with different functional additives, current research mostly focuses on the first category of strategies to improve ion transport and deposition behavior, optimizing kinetic performance through low-viscosity solvents or structural regulation. In contrast, research on additives that can form a stable SEI/CEI on the aluminum anode surface through controlled decomposition and participate in interfacial chemical regulation remains very limited, which also leaves important exploration space for the future design of functional electrolytes. Currently, some studies have added chlorides, such as lithium chloride, potassium chloride, and sodium chloride, into AlCl_3_/[EMIm]Cl electrolytes, utilizing these additives to form a more robust SEI protective layer on the aluminum metal anode surface, thereby suppressing parasitic side reactions at the negative electrode interface.^26,99^ The research in Passerini's group^99^ showed that when sodium chloride is introduced into the system as a model additive, a SEI layer of Na_*x*_Al_*y*_O_2_ with increased thickness is formed on the surface of the aluminum metal anode (Fig. 8a and b). The formation of this interface can regulate the aluminum deposition/dissolution process, inhibit dendrite generation, and help suppress the deposition of polysulfides on the anode. Similar results were also observed when using lithium chloride or potassium chloride as additives. After 40 cycles, the electrodes cycled in the AE-2Na electrolyte showed carbon, nitrogen, and chlorine signals derived from the deposition of electrolyte components and/or their decomposition products (Fig. 8c). Meanwhile, the signal peak produced by sodium atoms is particularly significant, showing that Na also appears in this SEI. Test results using the modified electrolyte show that the Al–S battery assembled with AE-2Na can obtain a specific discharge capacity of 473 mA g^−1^ after 50 charge/discharge cycles at 100 mA g^−1^ (Fig. 8d).

In the optimization of anode stability for rechargeable AIBs, Xie *et al.*^100,102^ introduced the cationic surfactant cetyltrimethylammonium chloride (CTAC) and the anionic surfactant sodium dodecylbenzene sulfonate (SDBS) as electrolyte additives in 2024 and 2025, respectively. By constructing dynamic adsorption layers on the electrode surface, they effectively inhibited aluminum dendrite growth and enhanced battery performance from the perspective of interfacial regulation. The CTAC molecules added to the electrolyte preferentially adsorb at the anode/electrolyte interface, which not only promotes a uniform aluminum electrodeposition/stripping process but also improves the wettability of the electrode/electrolyte interface through its amphiphilic molecular structure, thereby facilitating ion migration and diffusion (Fig. 8e). A stable anode/electrolyte interface reduces the local current density on the electrode surface and suppresses concentration polarization (Fig. 8f), leading to the significant inhibition of corrosion and dendrite growth on the aluminum metal anode. Benefiting from the interfacial optimization by CTAC, the assembled symmetric cells can cycle stably for over 1200 h at 3 mA cm^−2^ and 1 mAh cm^−2^. The Al//FG full cells exhibit outstanding rate performance and cycling stability, with a 10.5% increase in specific capacity after 600 cycles. Unlike CTAC, which regulates ion kinetics and deposition processes *via* the positive charge of the quaternary ammonium cation attracting anions, SDBS exerts these effects through the electrostatic shielding of the anionic –SO_3_^−^ group. Specifically, the surface activity of the alkyl chain in SDBS promotes its preferential adsorption on the electrode surface, forming a protective molecular interfacial layer that mitigates the corrosion of the aluminum metal anode by chloroaluminates (Fig. 8g). Meanwhile, the moderate electronegativity of the phenyl –SO_3_^−^ group generates an electrostatic shielding effect, which not only dynamically regulates ion migration but also adjusts nucleation and deposition rates, achieving smooth and uniform aluminum electrodeposition (Fig. 8h–j). Al‖Al symmetric cells assembled with SDBS-containing electrolytes achieve 500 h of cycling stability at a high current density (5 mA cm^−2^) and maintain stable cycling for up to 1000 h at a large areal capacity (5 mAh cm^−2^).

While the incorporation of functional additives significantly enhances the ion transport kinetics and interfacial stability of conventional IL electrolytes, these benefits are often achieved at the expense of increased formulation complexity and do not address the intrinsic high cost of imidazolium-based salts. Moreover, additives remain dissolved species within the liquid matrix and cannot fundamentally alter the moisture sensitivity or the thermodynamic instability inherent to liquid systems. Consequently, to better balance electrochemical performance and economic viability, deep eutectic solvents (DESs) have emerged as an alternative electrolyte design strategy. By utilizing inexpensive hydrogen bond donors and acceptors, DESs offer a promising low-cost alternative to traditional ILs, which will be discussed in detail in the following section.

### 4.3. Eutectic solvent design for property optimization

When discussing ILs, attention should also be paid to various IL analogues or hybrids developed over the past decade. These materials typically possess properties like traditional ILs but exhibit subtle differences in structure or composition. They can be collectively referred to as IL analogues (ILAs), which belong to the fourth-generation IL systems.^49^ ILAs contain both ionic and neutral components, which interact through either hydrogen bonding, typically forming DESs, or Lewis acid–base interactions, giving rise to liquid coordination complexes or solvated ILs.^142–144^ In terms of physical properties such as melting point, vapor pressure, and non-flammability, the differences between DESs and ILs are not significant. However, the interaction mechanisms and compositions at the molecular level are fundamentally different. In DESs, the ionic and molecular species are formed primarily through electrostatic forces and hydrogen bonding, whereas ILs rely almost exclusively on electrostatic interactions because they contain only ions and exhibit weak hydrogen bonding.^145^ Despite the commonalities among various electrolytes, ILs and DESs each have their own advantages and disadvantages due to differences in their initial components and mechanisms of action. For example, ILs exhibit lower viscosity because of their weak-to-moderate hydrogen bonding, while DESs possess higher viscosity due to their stronger hydrogen bonding.^146,147^

**Fig. 9:**
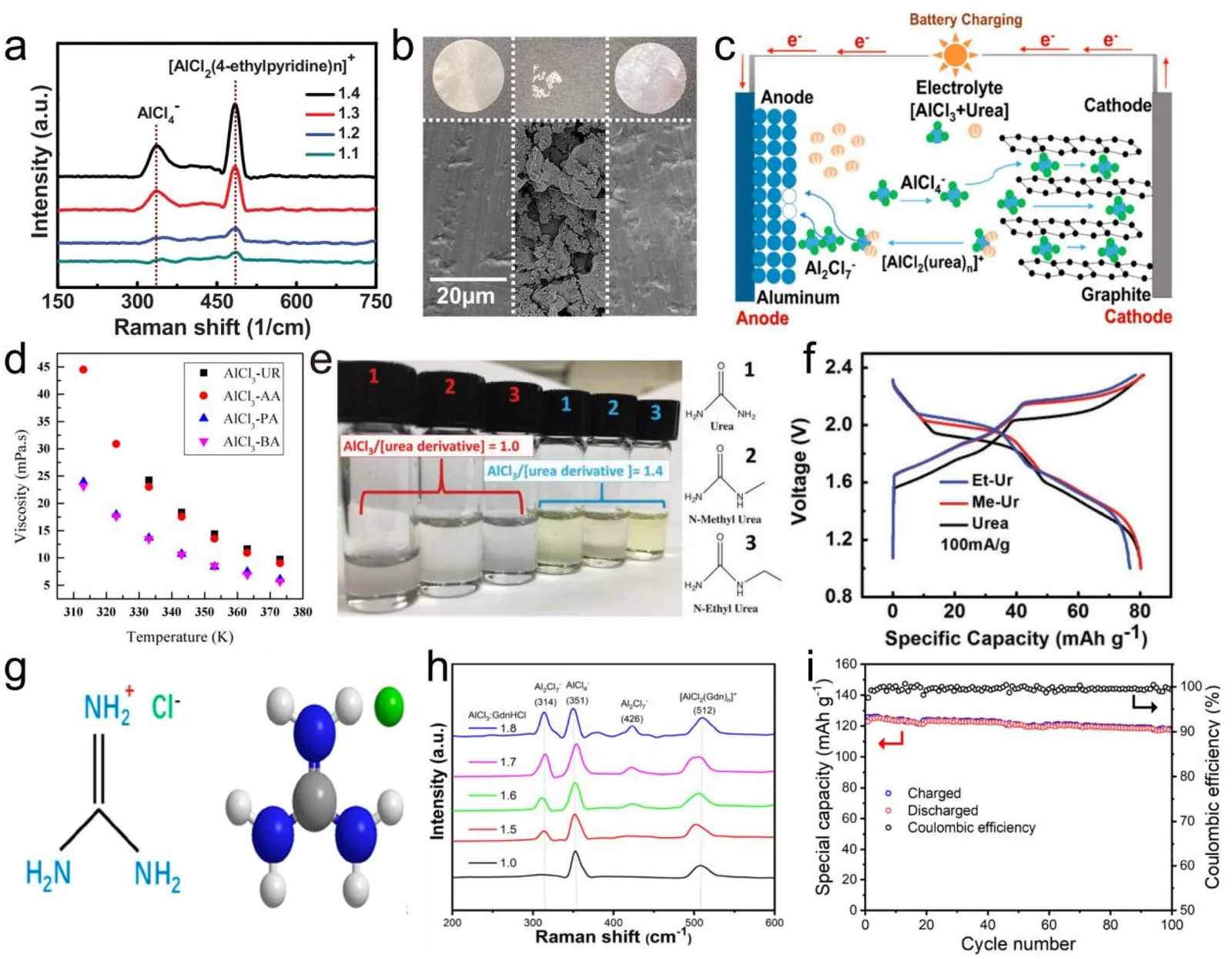
(a) Raman spectra of IL electrolytes with different AlCl_3_/4-ethylpyridine molar ratios. (b) Al foil before (left) and after being immersed in traditional IL (center) and 4-ethylpyridine–AlCl_3_ IL (right) at 60 °C for 10 days. Reproduced with permission.^106^ Copyright 2020, Wiley-VCH. (c) Diagram of battery charging with Al deposition and anion intercalation in graphite. Reproduced with permission.^104^ Copyright 2017, Proc. Natl. Acad. Sci. (d) Effect of temperature on the viscosity of AlCl_3_-amide IL analogues. Reproduced with permission.^152^ Copyright 2017, Elsevier. (e) Urea-based compounds and the related ILA electrolytes. (f) Galvanostatic charge/discharge curves at 100 mA g^−1^ for Al batteries using AlCl_3_/[urea derivative] = 1.4. Reproduced with permission.^105^ Copyright 2020, Wiley-VCH. (g) Chemical structure of the guanidine hydrochloride. (h) Raman spectra of AlCl_3_/GdnHCl electrolytes with different molar ratios. (i) Cyclic stability of the full cell at 0.1 A g^−1^. Reproduced with permission.^107^ Copyright 2023, American Chemical Society.

Due to their low cost, simple preparation, environmental friendliness, chemical stability, and excellent electrochemical performance, DESs are regarded as potential alternative electrolytes for AIBs, belonging to the class of low-cost ILAs. The choice of ligands directly determines the physicochemical properties and chemical composition of DESs. DESs share anion types with ILs (such as Al_2_Cl_7_^−^ and AlCl_4_^−^), but the cations vary depending on the ligands (AlCl_2_·(ligand)_*n*_^+^). In addition to traditional Al_2_Cl_7_^−^, special cations formed by certain ligands, such as [AlCl_2_·(AcA)_2_]^+^ and [AlCl_2_(4-ethylpyridine)_2_]^+^, can also support the reversible electrodeposition/dissolution of aluminum. For example, Li *et al.*^106^ proposed using AlCl_3_ and 4-ethylpyridine in a 1.3 molar ratio to construct a eutectic electrolyte. This system does not contain Al_2_Cl_7_^−^, and its [AlCl_2_(4-ethylpyridine)_2_]^+^ species serves as the anodic redox-active species (Fig. 9a). Compared to traditional ILs, this eutectic system exhibits significantly reduced corrosivity toward aluminum, copper, and nickel electrodes (Fig. 9b) and is insensitive to moisture, which facilitates the assembly and application of AIBs. Furthermore, the assembled full cells achieved a high capacity retention of 75% after 100 cycles. Although cation-assisted Al-anode reactions often involve high dissociation energy barriers, they offer significant advantages in two aspects: on the one hand, cation-dominated reduction reactions can avoid kinetic hindrance caused by opposing migratory and diffusive forces. On the other hand, the absence of Al_2_Cl_7_^−^ enhances the moisture and corrosion resistance of the system.^148^

In 2017, Angell *et al.*^104^ conducted the pioneering work of synthesizing the inaugural eutectic electrolyte for AIBs by blending AlCl_3_ and urea at a 1.3 : 1 molar ratio (Fig. 9c). While this AlCl_3_-urea archetype supports reversible Al plating/stripping, its practical efficacy is hindered by significant rheological constraints, sluggish ionic transport, and a restricted electrochemical stability window (≤2.35 V). Consequently, recent research has pivoted toward the integration of diverse organic ligands to engineer eutectic architectures with optimized physicochemical profiles and expanded anodic stability.

Viscosity is one of the most critical physical properties of eutectic electrolytes, which can be defined as the ability of a liquid to resist deformation under a specific shear rate. In practice, high-viscosity liquids flow slowly and uniformly, while low-viscosity liquids flow more easily, which has a significant impact on ion transport in batteries. Research has found that AlCl_3_-based eutectic electrolytes typically exhibit high viscosity at room temperature. For example, AlCl_3_ : urea electrolyte is 89 mPa s and AlCl_3_ : acetamide (AA) electrolyte is 70 mPa s. The phenomenon of high viscosity in eutectic electrolytes can be explained by the hole theory, which originates from the limited internal free volume within the liquid.^149–151^ The presence of a large number of neutral and undissociated AlCl_3_-ligand complexes in the electrolyte leads to an increase in viscosity, a decrease in conductivity, and slower reaction kinetics in AIBs. One feasible method to reduce viscosity is to replace protons in the ligand with polar groups, thereby disrupting the hydrogen-bonding network, reducing viscosity, and enhancing ionic conductivity.^148^ Liu *et al.*^152^ studied the effects of temperature, molar ratio, and different urea derivatives on the viscosity of AlCl_3_-based DESs. At the same molar ratio, the viscosity of these eutectic electrolytes increases in the following order: butyramide < propionamide < acetamide < urea, which is related to the variable hydrogen bonding between amides and aluminum complexes. Generally, the total hydrogen bonds formed between hydrogen bond donors and acceptors significantly affect the viscosity of eutectic electrolytes. Since urea carries two amino groups, this usually leads to the formation of two hydrogen bonds, whereas other urea derivatives typically carry only one amino group, which reduces the number of hydrogen bonds in the electrolyte.^105,153^ Moreover, studies found that the viscosity of AlCl_3_ : urea derivatives follows Arrhenius-type behavior, which decreases as temperature increases (Fig. 9d). In 2019, Angell *et al.*^105^ systematically investigated the performance changes of *N*-methylurea (Me-Ur) and *N*-ethylurea (Et-Ur) as ligands to construct ILAs with AlCl_3_ (Fig. 9e). Experimental results showed that the introduction of these two urea derivatives significantly reduced the electrolyte viscosity, with the AlCl_3_/Et-Ur (molar ratio is 1.4) system exhibiting a viscosity of only 45.0 cP, while its ionic conductivity increased to 1.56 mS cm^−1^. Benefiting from the sharp reduction in viscosity and the simultaneous improvement in conductivity, the battery with AlCl_3_/Et-Ur achieved a high discharge plateau voltage of 2.08 V at 100 mA g^−1^, effectively improving the energy density of the battery (Fig. 9f). This result confirms that simple alkylative modification of urea molecules can significantly optimize the kinetic performance and output characteristics of AIBs without sacrificing CE.

Besides viscosity, ionic conductivity is another important characteristic of eutectic electrolytes worthy of attention in battery applications, as it characterizes the ions’ migration ability in the electrolyte.^146^ The intrinsically low ionic conductivity of eutectic electrolytes can also be explained by the hole theory, which posits that ionic movement is limited by the viscosity and ionic size of the liquid system. According to this theory, voids or holes in the eutectic electrolyte at low temperatures limit the free movement of ions, leading to a decrease in conductivity, primarily due to the inhibition of ion migration by higher viscosity.^151^ To improve the conductivity of eutectic electrolytes, several measures can be taken. First, using smaller cations or replacing hydrogen bond donors with fluorides can increase the voids or free volume in the system, thereby improving ionic mobility. In addition, adjusting factors such as hydrogen bond acceptors, the alkyl chain length of cations, and temperature can effectively regulate the conductivity of eutectic electrolytes. Among these factors, the influence of temperature changes on conductivity is particularly significant. Increasing temperature typically disrupts the original hydrogen-bonding network, thereby promoting ion migration and significantly increasing the ionic conductivity of the eutectic electrolyte. Another comparative study of mixtures of AlCl_3_-based electrolytes with amide molecules, such as acetamide, propionamide, and butyramide, indicates that intermolecular electrostatic interactions and molecular size significantly influence ionic conductivity.^154^ As the molecular size of the ligand increases from acetamide to propionamide and then to butyramide, the conductivity of the electrolyte exhibits a trend of first increasing and then decreasing. Kang *et al.*^107^ constructed an AlCl_3_/guanidine hydrochloride (GdnHCl) eutectic electrolyte (Fig. 9g and h) and utilized the synergistic coordination effect of the three nitrogen groups in the GdnHCl molecule to significantly enhance the carrier concentration and migration efficiency within the system, resulting in a maximum ionic conductivity of 10.57 mS cm^−1^. Based on this, the Al–graphite battery achieved a high capacity of 128 mAh g^−1^ at 100 mA g^−1^ (Fig. 9i) and maintained a CE of nearly 99.5% after 1000 stable cycles at 1 A g^−1^.

**Fig. 10:**
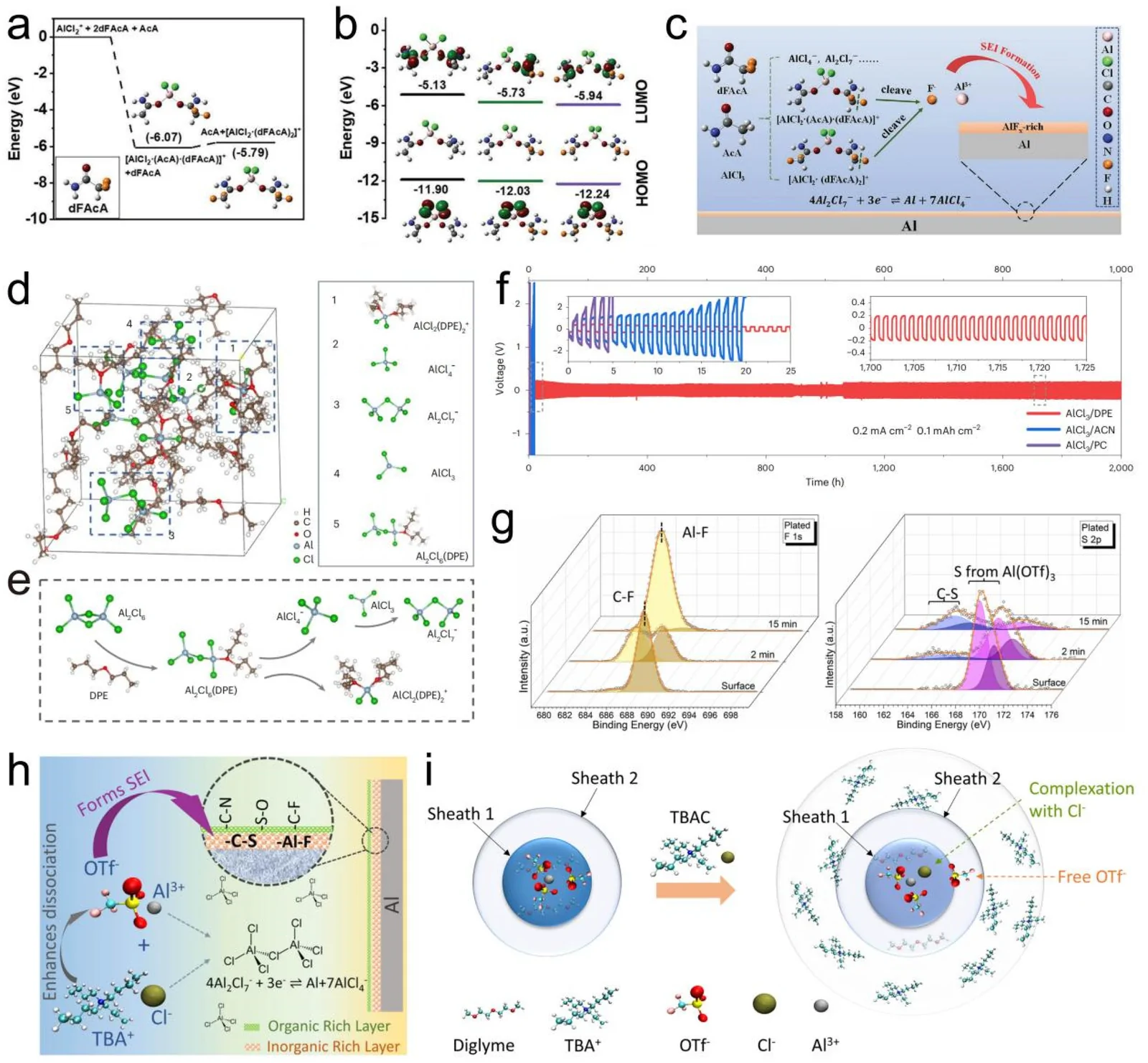
(a) Energy profiles of dFAcA coordination with AlCl_2_^+^*via* carboxyl O atom. (b) Calculated HOMO and LUMO for different coordinated cations. (c) Schematic illustration of the process steps. Reproduced with permission.^109^ Copyright 2024, Wiley-VCH. (d) Snapshot of an AIMD-simulated cell for the AlCl_3_/DPE electrolyte. (e) Schematic illustration of the solvation interactions between Al_2_Cl_6_ and DPE molecules. (f) Galvanostatic aluminum plating/stripping curves in symmetric cells at 0.2 mA cm^−2^. Reproduced with permission.^110^ Copyright 2026, Springer Nature. (g) F 1s and S 2p XPS spectra for the plated aluminum foil. (h) Schematic illustration of the process steps of electrolyte modification and the formation of SEI layer. (i) Illustration of Al-ion solvation sheath changes following TBAC incorporation into Al(OTf)_3_–diglyme electrolyte. Reproduced under terms of the CC-BY license.^159^ Copyright 2023, Springer Nature.

Eutectic electrolyte systems based on urea and acetamide exhibit a relatively limited ESW (usually ≤2.35 V). The ESW of the electrolyte has a critical impact on the energy density and durability of the battery. If the working voltage of the battery exceeds the range of the electrolyte's ESW, the cycling efficiency will be significantly affected. Therefore, electrolytes with a wider ESW are crucial for battery performance. However, there are relatively few research reports on the electrochemical stability window of eutectic electrolytes. Yu's group^109^ found that by introducing difluoroacetamide (dFAcA) as an additive in the AlCl_3_/acetamide (AcA) electrolyte system, the coordination number of AcA with aluminum chloride was successfully reduced due to the electron-withdrawing effect of fluorine. This increased the oxidation potential from 2.21 V to 2.78 V (*vs.* Al^3+^/Al). Further research showed that the [AlCl_2_·(AcA)·(dFAcA)]^+^ and [AlCl_2_·(dFAcA)_2_]^+^ compounds possess lower HOMO levels, exhibiting enhanced antioxidant capacity, extended oxidation potential, and improved high-voltage stability (Fig. 10a and b). Additionally, these compounds can stabilize the electrode interface by forming an AlF_*x*_ layer *in situ* on the aluminum electrode surface, which significantly reduces the interfacial resistance (Fig. 10c). Aluminum-sulfur batteries containing the dFAcA additive exhibit a stable discharge plateau voltage in the 1.7–1.9V range, and the battery performance showed no significant decay after 300 cycles.

One of the main problems facing traditional chloroaluminate ILs in practical applications is their strong corrosivity. These ILs contain various complex chloroaluminate species such as Al_2_Cl_7_^−^, and these highly reactive components are generally considered the core reason for the corrosion of metal substrates. Although there have been many research reports on the corrosion behavior caused by IL electrolytes, systematic research on the corrosion mechanism induced by eutectic electrolyte systems remains very limited, and the relevant literature is still a blank. In a recent study, Zhang *et al.*^110^ developed an organic chloride eutectic electrolyte based on AlCl_3_ and dipropyl ether (DPE). By leveraging the moderate solvation capability of DPE to trigger the asymmetric cleavage of Al_2_Cl_6_ dimers, they generated monovalent AlCl_2_(DPE)_2_^+^ cations. Crucially, the corrosive-free Cl^−^ species are entirely sequestered within the solvation sheath of these cations (Fig. 10d and e), providing robust protection for stainless steel current collectors and other cell components. Empirical results indicate that stainless steel foils maintain a pristine, pit-free surface even after 90-day immersion, with the corrosion current density plummeting by two orders of magnitude relative to conventional ILs. This electrolyte sustains stable Al plating/stripping for over 2000 h at 0.2 mA cm^−2^ (Fig. 10f). The Al//Mo_6_S_8_ full cells delivered an impressive 75% capacity retention after 300 cycles, showcasing the dual merits of corrosion mitigation and electrochemical performance optimization.

In recent years, chloride-free electrolytes have received widespread attention, aiming to avoid the critical limitations brought by corrosive chloroaluminate systems. However, the passive films formed on aluminum anodes in chloride-free eutectic electrolytes cannot be removed effectively, thereby hindering reversible aluminum deposition/dissolution.^155–158^ To solve the problems of irreversible aluminum plating/stripping and low CE in such electrolytes, Seh *et al.*^159^ proposed a novel electrolyte system based on Al(OTF)_3_: by introducing 0.1 M tetrabutylammonium chloride into diethylene glycol, reversible aluminum deposition/dissolution behavior was achieved. Instead of relying on corrosive chloroaluminates, this system generates Al_2_Cl_7_^−^ and AlCl_4_^−^ active species *in situ* through trace chlorine-containing additives, thereby promoting the breakdown of the oxide film and electrochemical activation. Meanwhile, the OTF^−^ anions released by the system can form a solid electrolyte interphase during decomposition. Its organic components are mainly composed of S–O, C–F, and C–N bonds, while the inner inorganic phase is rich in C–S and Al–F components, effectively protecting the aluminum anode surface and inhibiting further oxidation (Fig. 10g–i).

Eutectic solvents offer a cost-effective pathway, yet their relatively low ionic conductivity and unclear corrosion mechanisms limit their application in high-performance devices. Crucially, although ligands such as urea or acetamide are generally regarded as relatively low-hazard components, the incorporation of AlCl_3_ introduces important environmental trade-offs. The inherent moisture sensitivity of chloroaluminate species poses risks of hydrolysis, potentially releasing corrosive HCl that threatens both manufacturing safety and ecosystem integrity. Consequently, the environmental advantages of DESs depend not only on their molecular components but also on appropriate handling and containment throughout their lifecycle. To address these challenges, future research should focus on developing biodegradable ligands that preserve anodic reversibility while reducing environmental impacts. Ultimately, combining the ionic transport advantages of liquid electrolytes with the mechanical robustness of solid-like systems represents a promising direction for the development of gel and quasi-solid-state electrolytes.

### 4.4. Gel and quasi-solid architecture construction for solid-state transition

In traditional battery manufacturing, liquid electrolyte systems (including ILs, organic electrolytes, and aqueous electrolytes) generally suffer from issues such as insufficient electrochemical performance, low safety, and poor stability. Specifically, IL electrolytes are typically corrosive, flammable, and toxic, and are prone to gas emissions and liquid leakage during charge–discharge processes.^160–163^ In contrast, polymer electrolytes have gradually become a research hotspot due to their advantages of high safety, good flexibility, and multifunctionality. More importantly, polymer electrolytes can not only effectively solve liquid leakage problems but also eliminate the reliance on separators to prevent short circuits, simultaneously serving as ion transport media and physical isolation layers to simplify battery structures.^164–167^ Currently, polymer electrolytes for non-aqueous AIBs mainly focus on two forms: solid polymer electrolytes (SPEs) and gel polymer electrolytes (GPEs). SPEs contain no liquid components and possess excellent thermal stability and mechanical strength, but their room-temperature ionic conductivity is low. While GPEs, as semi-solid gels between liquid and solid states, combine high ionic conductivity with good mechanical properties, making them more suitable for practical applications under existing conditions.

The two key factors of polymer electrolyte performance—mechanical strength and ionic conductivity—are closely related but often contradictory. High mechanical strength helps maintain the structural integrity of separator-free designs and prevents dendrite penetration, while high ionic conductivity enhances ion migration kinetics. Typically, increasing the polymer framework content enhances mechanical strength but limits segmental motion, thereby hindering ion transport. Conversely, increasing the concentration of ILs or eutectic electrolytes favors ionic conductivity but weakens the mechanical support. To resolve this contradiction, current optimization strategies mainly include framework structural modification (such as cross-linking density control, nanocomposites) and solvation structure design (such as polymer ionization, competitive coordination strategies), aiming to achieve the synergistic enhancement of ion transport efficiency and physicochemical stability.^148^ For example, solvent-guided hierarchical network formation can construct SPEs with both high strength and high ionic conductivity, while competitive coordination strategies can decouple mechanical strength and conductivity, enabling gel electrolytes to achieve high toughness and fast ion transport simultaneously.

Compared to traditional liquid electrolytes, SPEs provide significantly enhanced safety guarantees for battery systems due to their superior thermal stability, non-flammability, and excellent chemical stability. This high safety originates not only from the flame-retardant properties of the materials themselves but also because they can serve as monolithic components that simultaneously fulfill the functions of ion transport and physical separation, effectively preventing direct contact between the positive and negative electrodes.^168,169^ At the electrode interface, SPEs typically exhibit lower side-reaction activity, which helps construct electrochemical systems with lower internal resistance, higher power output, and better cycling durability. However, despite these obvious advantages, the application of SPEs in AIBs still faces severe scientific challenges. Current research is still in its early stages. The core issue is that the high charge density of trivalent aluminum ions triggers extremely strong coulombic interactions, while the large ionic radius and volume effects of chloroaluminate anions significantly increase their migration resistance in dense polymer media. Furthermore, the high content of ILs introduced to ensure ionic conductivity often weakens the mechanical protection of the polymer skeleton, leading to problems such as gas evolution and the loss of active species.^170–173^ These factors collectively restrict effective ion transport in solid-state aluminum-ion electrolytes and have become the main bottlenecks for improving overall battery performance.

**Fig. 11:**
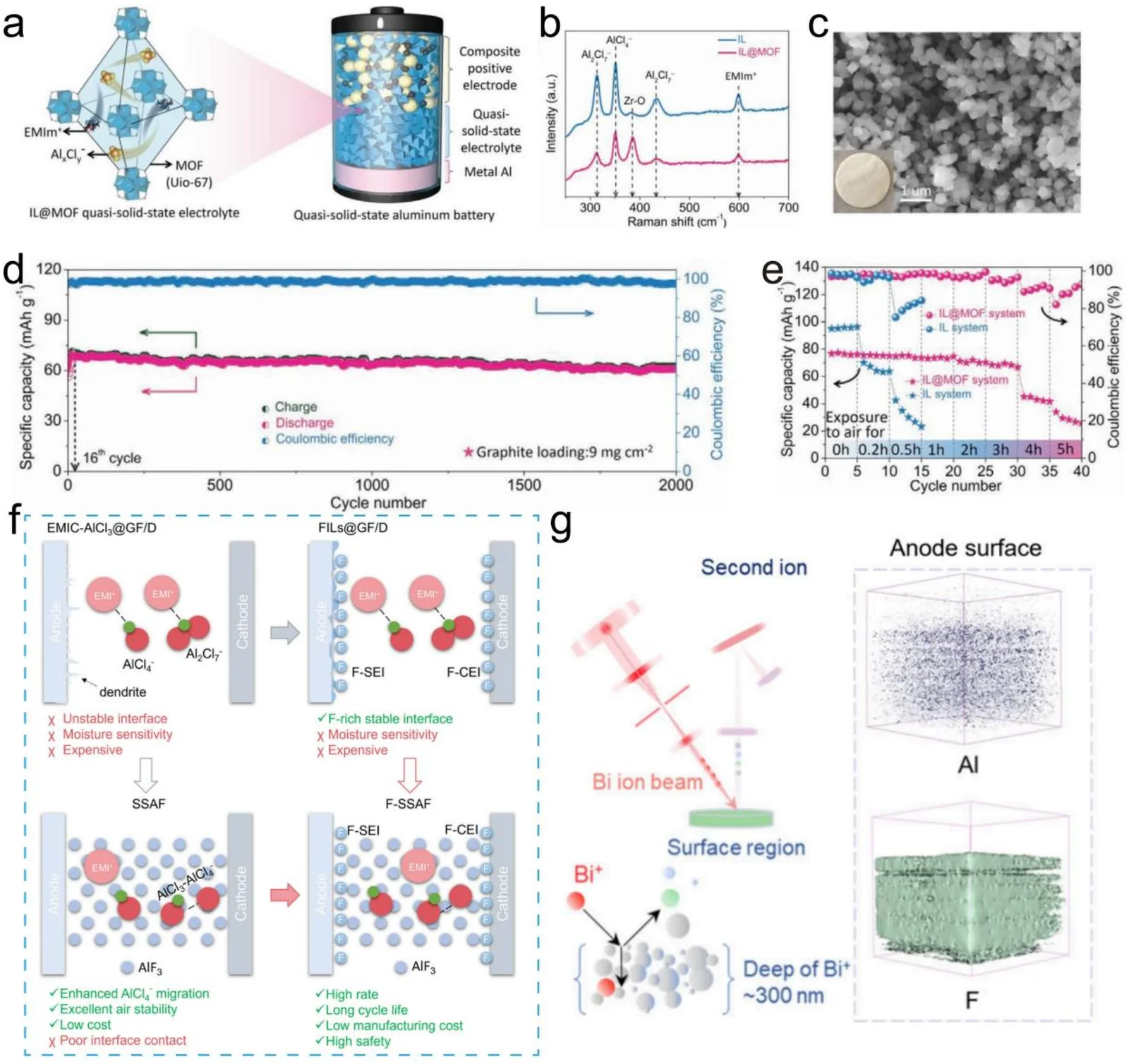
(a) Schematic representation of the internal structures of the IL@MOF electrolyte and the design of the quasi-solid-state aluminum cell. (b) Raman spectra of different electrolytes. (c) SEM morphology of IL@MOF particle. (d) Cycling stability of the Al|IL@MOF|C full cell. (e) Specific discharge capacity and CE of full cells after exposing in air for different time scans. Reproduced with permission.^111^ Copyright 2022, Wiley-VCH. (f) Schematic illustration of the AIBs using different electrolytes. (g) ToF-SIMS 3D reconstruction of the sputtered volume from the Al anode surface, with a schematic illustration. Reproduced with permission.^112^ Copyright 2025, American Chemical Society.

As early as 2022, Jiao's group^111^ constructed an IL@MOF quasi-solid-state electrolyte by encapsulating ILs within a zirconium-based metal–organic framework (UiO-67) (Fig. 11a–c), achieving high ionic conductivity, a wide electrochemical window, and excellent air stability. The porous framework of the MOF confines the IL within its channels, forming a continuous ion transport network between the electrode and electrolyte while blocking moisture from the air, thereby significantly improving the air stability and safety of the electrolyte. Benefiting from the stable electrode/electrolyte interface, the quasi-solid-state Al battery based on this electrolyte maintained a capacity of approximately 62 mAh g^−1^ after 2000 cycles at 100 mA g^−1^, with a CE of about 98% (Fig. 11d). As shown in Fig. 11e, this quasi-solid-state electrolyte maintained good electrochemical performance even after several hours of exposure to air, showing good practical prospects. Subsequently, in 2025, Jiao and his co-workers^112^ proposed a solid-state electrolyte based on an AlF_3_ inert inorganic framework (F-SSAF). This design utilizes AlF_3_ to promote the dissociation of Al_2_Cl_7_^−^, increasing the anion transference number to 0.50 and significantly inhibiting the growth of Al dendrites. Furthermore, when FEC@EMIC-AlCl_3_ (FIL) was introduced into the electrolyte system as an additive, a fluorine-rich SEI layer was formed *in situ* on the anode surface, thereby regulating the ion transport/deposition kinetics at the anode/electrolyte interface and inhibiting the corrosion of the aluminum anode (Fig. 11f and g). Symmetric cells based on this solid-state electrolyte achieved ultra-long stable Al deposition and dissolution for up to 4000 h. Full cells achieved an ultra-long life of 10 000 cycles and an average CE of >99%. More importantly, this study achieved an 80% high recovery rate of the inorganic framework in solid-state Al batteries for the first time, providing a new approach for the practical application of cost-effective solid-state Al batteries.

Broadening the ESW of IL electrolytes to −2 V *vs.* Al/Al^3+^ is a key prerequisite for stabilizing [EMIm]^+^ cations and driving the reversible deposition of chloroaluminate ions. Achieving this electrochemical window can inhibit the decomposition of the electrolyte, significantly enhancing the specific energy and energy density of the battery by reducing the ineffective consumption of chloroaluminate species.^174^ However, although SPEs are a solution to multiple issues related to liquid electrolytes, including those mentioned above, low ionic conductivity at room temperature remains the main weakness of SPEs. Therefore, how to achieve this goal while maintaining high ionic conductivity remains a core challenge. A very promising strategy is to introduce polymers to construct gel networks, where a small amount of non-reactive polymers (such as poly(propylene oxide) (PPO), poly(methyl methacrylate) (PMMA), or poly(vinylidene difluoride) (PVDF)) entangle with IL molecules to give the electrolyte a rubbery or gel-like macroscopic morphology, thereby effectively suppressing leakage and improving safety.^175–177^ In this context, Dai *et al.*^178^ reported the first AIBs based on a gel polymer electrolyte in 2015. Their innovation lay in using radical polymerization methods with aluminum chloride-complexed acrylamide as a functional monomer, combined with an acidic composition of [EMIm]Cl and AlCl_3_, successfully preparing a GPE that possesses both good mechanical properties and ion transport capacity, laying an important foundation for the subsequent development of solid-state aluminum batteries.

To address the persistent trade-off between mechanical robustness and room-temperature ionic conductivity in conventional SPEs, research has increasingly shifted towards IL-based gel electrolytes. By leveraging a polymerized network to immobilize ionic liquids, these electrolytes integrate the high safety of solids with the superior ion transport kinetics of liquids. This unique combination, along with inherent merits such as non-flammability, good thermal stability, and wide electrochemical windows, has driven their rapid development. Consequently, gel electrolytes can improve battery safety by immobilizing potentially hazardous ionic liquid components within a polymer matrix, thereby preventing leakage and reducing the risk of environmental release. This architectural confinement aligns with the *Green Chemistry* principle of designing safer chemicals, although the limited recyclability of the composite polymer matrix remains a challenge for end-of-life management.

**Fig. 12:**
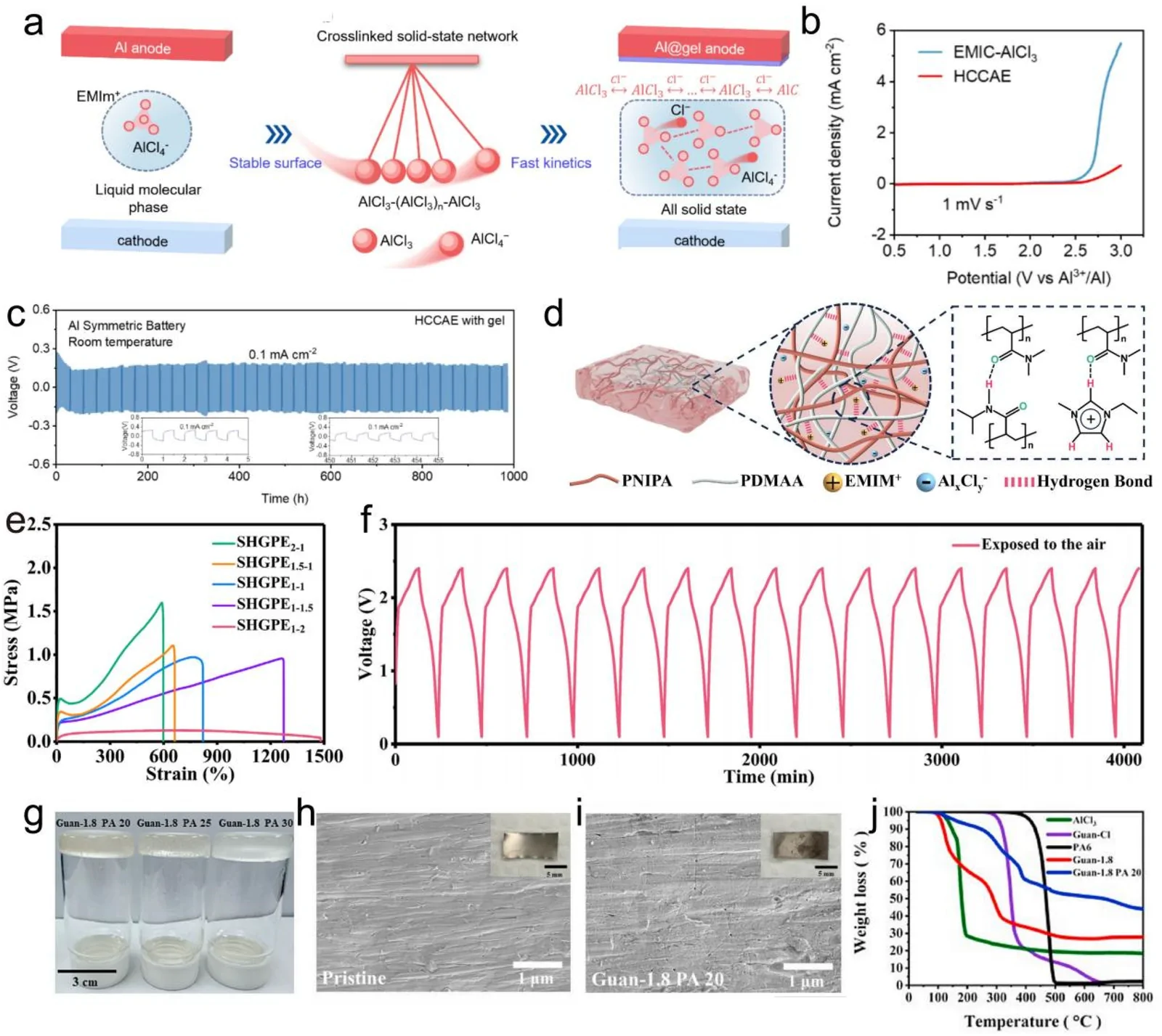
(a) Schematic depiction of electrolyte transformation: from liquid electrolyte to solid-state composite layer. (b) LSV curves of the Al‖Mo cells with different electrolytes. (c) Galvanostatic aluminum deposition/dissolution curves of symmetric cells at 0.1 mA cm^−2^. Reproduced with permission.^179^ Copyright 2025, American Chemical Society. (d) Illustration of hydrogen-bonding interactions within SHGPE. (e) Stress–strain curves of SHGPEs prepared with varying molar ratios of NIPA and DMAA. (f) The charge/discharge curve of the unsealed pouch cell. Reproduced with permission.^116^ Copyright 2026, Wiley-VCH. (g) Photographs of PAGPEs prepared with different amounts of PA6 in Guan-1.8. SEM images of Ni foil (h) before and (i) after being immersed in Guan-1.8 PA 20 for 40 h. (j) TGA curves of different electrolytes. Reproduced with permission.^63^ Copyright 2026, Elsevier.

In a recent representative work, Guo *et al.*^179^ developed a hyper-coordinated chloroaluminate electrolyte (HCCAE) based on a chain-assisted ion transport mechanism using aluminum chloride-complexed acrylamide as a functional monomer and acidic EMIC-aluminum chloride as a plasticizer. This study broke through the limitations of traditional ILs relying on discrete AlCl_4_^−^ and Al_2_Cl_7_^−^ ion conduction. By regulating the AlCl_3_/EMIC molar ratio (≥2 : 1), a long-range chain-like network structure characterized by [AlCl_3_-(AlCl_3_)_*n*_-AlCl_3_-AlCl_4_^−^] was constructed, forming a localized exchange-type ion transport path like a Newton's cradle (Fig. 12a). MD simulations and electrostatic potential calculations further revealed the chloro-bridging-induced hyper-coordinated structure and its promoting effect on ion transport kinetics, providing a new theoretical perspective for anion-dominated ion transport mechanisms in solid-state aluminum batteries. The HCCAE electrolyte designed based on this achieved a high ionic conductivity of 0.89 mS cm^−1^ at room temperature, a wide electrochemical window of >2.6 V (Fig. 12b), and a high anion transference number of 0.46. Al‖Al symmetric cells exhibited stable Al plating/stripping behavior for over 900 hours (Fig. 12c).

In addition, Li *et al.*^180^ proposed and verified a novel battery structure based on a nitrogen-doped carbon ionic/electronic dual-conductive interlayer (NCIL) for gel electrolytes. By introducing NCIL between the gel polymer electrolyte and the CuSe cathode, the ion transport characteristics can be regulated by increasing the anion transference number, thereby promoting reaction kinetics and suppressing Al dendrite proliferation, significantly extending the cycle life of the battery. As another proof of concept, Fu's group^116^ constructed a self-healing gel polymer electrolyte (SHGPE) with a dynamic hierarchical hydrogen-bonding network through the solvent-free UV copolymerization of *N*-isopropylacrylamide (NIPA) and *N*,*N*-dimethylacrylamide (DMAA) in chloroaluminate ILs (Fig. 12d). As shown in Fig. 12e, this electrolyte utilizes reversible hydrogen bonding between polymer chains and between polymers and [EMIM]Cl to endow the material with excellent self-healing ability and mechanical properties (breaking stress 0.98 MPa, breaking strain 1275%). It significantly inhibits Al anode corrosion by restricting IL leakage and moisture erosion. Al symmetric cells based on this SHGPE achieved stable Al deposition/dissolution cycles for over 8000 h (about 333 days) at 0.5 mA cm^−2^, while Al‖graphite full cells showed an ultra-long life of 50 000 cycles at a high rate of 2 A g^−1^. Importantly, even after the electrolyte was cut and healed, the assembled battery could still cycle stably for over 4000 minutes (Fig. 12f), verifying the key role of the dynamic hydrogen-bonding network in repairing microscopic cracks, stabilizing the electrode/electrolyte interface, and suppressing dendrite growth, providing a feasible strategy for the design of flexible, long-life solid-state aluminum batteries.

Besides their widespread application in enhancing the electrochemical performance of conventional ILs, gel electrolytes have also been successfully introduced into ILAs/DESs systems. By constructing a three-dimensional network structure, they significantly enhance the mechanical strength and environmental stability of the electrolyte. Simultaneously, they utilize specific interactions between polymer segments and ions to regulate the solvation structure and interfacial chemistry, thereby effectively inhibiting dendrite growth, reducing interfacial impedance, and broadening the operating temperature range, providing a key solution for the application of eutectic electrolytes in high-safety, long-life energy storage devices. In 2026, Tsai *et al.*^63^ developed a polyamide-based gel polymer electrolyte (PAGPE) with excellent moisture resistance by introducing polyamide (PA6) into an AlCl_3_/guanidine hydrochloride ILA (Fig. 12g). This electrolyte utilizes the hydrogen bonding and coordination effects between polyamide and IL to regulate the ratio of Al_2_Cl_7_^−^, thereby significantly improving the corrosion resistance toward nickel current collectors (Fig. 12h and i) and thermal stability (with decomposition temperature rising to 120 °C) (Fig. 12j), while maintaining a high ionic conductivity of 5.25 mS cm^−1^. Al–graphite batteries based on this PAGPE achieved a specific capacity of approximately 125 mAh g^−1^ and a CE of nearly 99%.

## 5. Summary and future perspectives

AIBs have emerged as strong competitors for next-generation large-scale electrochemical energy storage systems (ESS) due to the high crustal abundance of aluminum, excellent theoretical volumetric capacity, and inherent high safety. Among various components of AIBs, the electrolyte is not only the medium for ion transport but also the core bottleneck determining the redox mechanism, interfacial stability, and overall electrochemical performance. Room-temperature chloroaluminate ILs, represented by AlCl_3_-[EMIm]Cl, have played a pioneering role in the development of AIBs. Possessing extremely low volatility, wide electrochemical stability windows, and non-flammable characteristics, they successfully realized the reversible deposition/stripping of aluminum at room temperature and fundamentally avoided the severe hydrogen evolution reaction in aqueous electrolytes. However, traditional ILs expose non-negligible shortcomings in commercial applications. First, the high synthesis cost of organic cation precursors, such as imidazolium salts, weakens the economic advantage of AIBs. Second, these electrolytes are highly moisture-sensitive and corrosive, and can release toxic HCl gas upon exposure to water, imposing stringent requirements on battery packaging and current collectors. Finally, high viscosity at room temperature leads to sluggish transport kinetics of chloroaluminate anions, making it difficult to meet the requirements of high-rate charging and discharging.

To overcome these challenges, researchers have conducted extensive studies on electrolyte modification and structure design, which can be summarized into four strategies, each with distinct advantages and disadvantages:

Anion−cation engineering: This strategy breaks the symmetry of traditional imidazolium ILs by altering ionic species or introducing cheap ligands, significantly improving oxidative stability and reducing costs. While acidic chloroaluminates enable reversible Al redox, their inherent corrosivity and moisture sensitivity limit material choices for current collectors and casings. Non-chloride or weakly acidic systems often fail to remove the native Al_2_O_3_ passive film, necessitating complex pre-activation.

Electrolyte additive engineering: As a cost-effective approach, introducing organic solvents or surfactants optimizes ion transport and deposition behavior. Specific additives can facilitate the construction of robust SEI/CEI layers to suppress parasitic reactions. However, most additives currently focus on physical regulation. Chemical-type additives capable of forming stable, long-term SEIs on Al anodes remain rare, and their long-term stability and side effects on cathodes require further verification.

Eutectic electrolytes: As green alternatives to ILs, these systems reduce costs and improve environmental friendliness *via* hydrogen-bonding networks. However, strong intermolecular interactions often lead to narrow ESW and high viscosity, limiting power density. Notably, systematic research on corrosion induced by deep eutectic solvents is still lacking, representing a significant gap.

Polymer electrolytes: To solve leakage and corrosion, polymer scaffolds provide enhanced safety and mechanical strength. Although solid-state systems suppress dendrites and side reactions, they suffer from high interfacial impedance. The strong coulombic interactions between Al^3+^ and the solid lattice result in extremely high migration energy barriers, making room-temperature conductivity insufficient. Reconciling high conductivity with mechanical strength remains a challenge, as does the long-term chemical stability of polymer skeletons in Lewis acidic environments.

As summarized in Table 2, these strategies involve distinct trade-offs rather than offering universal solutions. To provide a more actionable design framework, we now explicitly distill composition performance relationships to guide electrolyte selection. Chloroaluminate systems with high Al_2_Cl_7_^−^ concentrations favor high-rate reversible Al plating/stripping, but their intrinsic corrosivity necessitates the use of costly noble-metal current collectors. Conversely, chloride-free alternatives offer improved oxidation stability and moisture tolerance, but they generally require pre-activation to breach the Al_2_O_3_ passivation layer. Low-cost eutectic solvents and molecular diluents can reduce viscosity and material cost, although these benefits may be accompanied by narrowed electrochemical windows or compromised low temperature performance. Gel architectures improve safety through liquid immobilization, but they often introduce a trade-off between mechanical robustness and room-temperature ionic conductivity. Future electrolyte design should therefore balance these competing requirements by matching the formulation strategy, whether cation−anion engineering for reaction kinetics, additive incorporation for interfacial stability, DES formulation for cost reduction, or polymer architectures for safety, with the intended application and performance targets.

**Table 2:** Advantages and disadvantages of various strategies in AIBs with IL electrolytes

Strategy	Key advantages	Critical limitations
Cation–anion pair engineering	• Wide ESW	• High corrosivity (Cl-based)
	• Tunable physicochemical properties	• Passivation issues (Cl-free)
	• Low-cost options	
Functional additive incorporation	• Reduced viscosity	• Unverified long-term cycling stability
	• Enhanced ion transport	• Chemical SEI additives are rare
	• SEI regulation	
Eutectic solvent design	• Ultra-low cost	• High viscosity
	• Reduced corrosivity	• Narrow ESW
	• Green synthesis	• Unknown corrosion mechanisms
Gel/solid-state architecture	• Leakage-proof	• Low room-temp conductivity
	• High safety	• Poor interfacial contact
	• Mechanical robustness	

**Fig. 13:**
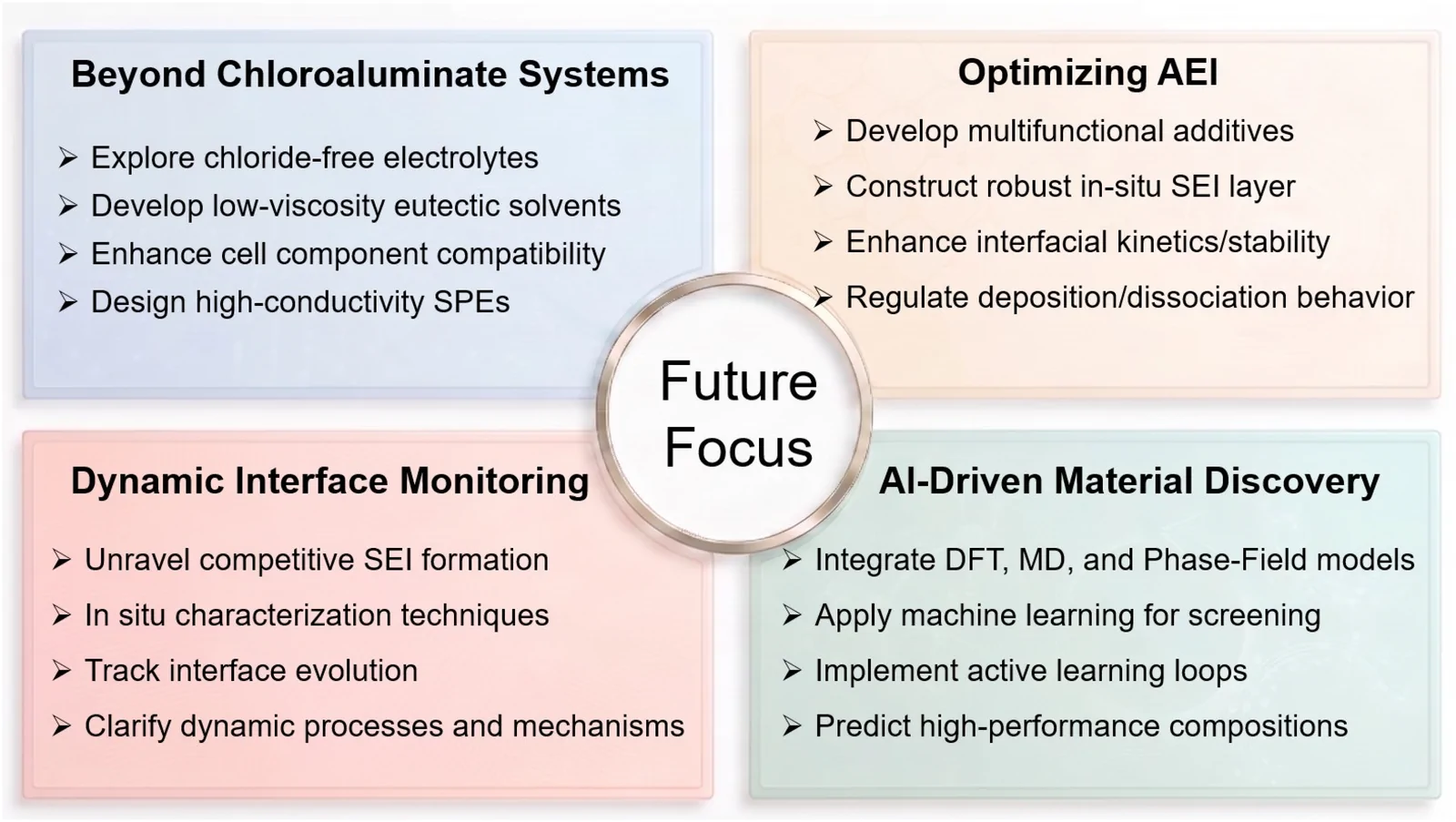
Future focuses on developing high-performance AIBs.

Although the research on AIBs and their electrolyte systems have made phased progress, many deep-seated scientific and technical challenges remain to bridge the gap from laboratory coin cells to large-scale engineering applications and achieve the ultimate goal of balancing high energy density, long cycle life, intrinsic safety, and low cost. Future research urgently needs to seek theoretical breakthroughs and technological innovations in the following four core dimensions (Fig. 13):

(1) To achieve high voltage and specific energy, future AIB research must move away from traditional chloroaluminate-based electrolytes. The focus should shift toward exploring novel liquid and solid systems that are chloride-free, operate across a wide temperature range, and are inherently non-corrosive. For liquid electrolytes, developing low-viscosity and environmentally friendly eutectic solvents is essential. This can be achieved by introducing large-volume anions with weak solvation effects, such as fluorinated or sulfonyl-based salts, to construct new ionic liquid systems that minimize corrosion and broaden the electrochemical window. Regarding solid polymer electrolytes, the primary goal is to improve the transport efficiency of multivalent Al ions within the solid network. This requires designing polymer backbones that weaken the coulombic attraction to Al ions by incorporating functional groups with large steric hindrance or weak coordination ability, while simultaneously increasing segment flexibility and free volume through copolymerization or cross-linking to facilitate ion hopping.

(2) The strong polarization of multivalent Al ions results in a high kinetic barrier for the dissociation process at the SEI. To address this, future research should focus on designing multifunctional electrolyte additives that regulate dissociation and deposition behavior. By precisely tuning the functional groups of cations and anions, these additives can weaken the electrostatic interactions within the solvation sheath, thereby lowering the dissociation energy barrier and guiding uniform aluminum nucleation. Moreover, harnessing the distinct hydrogen bonding and coordination chemistry within eutectic systems serves as a driving force for the directed assembly of durable artificial SEI and CEI layers, effectively enhancing the physicochemical compatibility at the electrode–electrolyte interface. For solid-state electrolytes, optimizing the formulation, such as adjusting the ratio of active species and polymer segments, is essential to improve the physicochemical compatibility between the electrode and solid matrix. This enhances interfacial ion transport kinetics and stabilizes the electrode surface against parasitic reactions.

(3) The interfacial chemistry of aluminum anodes involves a complex competitive process where chloroaluminate anions etch the native oxide layer to activate the electrode while simultaneously corroding the fresh aluminum substrate. This dynamic competition between corrosion and precipitation blurs the definition of SEI chemical components and causes disordered Al deposition. To resolve this ambiguity, future research must establish multi-modal *in situ* monitoring systems. It is essential to utilize techniques such as *in situ* X-ray diffraction (XRD) and solid-state nuclear magnetic resonance (NMR) to track phase evolution, while employing high-resolution probes like *in situ* Raman spectroscopy, electrochemical atomic force microscopy (EC-AFM), cryogenic electron microscopy (Cryo-EM), and quartz crystal microbalance with dissipation (QCM-D) to directly observe transient morphological changes, mechanical properties, and ionic adsorption behaviors at the interface.

(4) Given the vast combinatorial space of electrolyte materials, traditional trial-and-error experimentation is inefficient and time-consuming. Future development must integrate theoretical calculations with data science to create a multi-scale simulation-guided electrolyte design. This involves linking DFT, MD, and phase-field models to connect electronic structures with macroscopic phenomena like dendrite growth. More importantly, introducing machine learning and artificial intelligence algorithms enables high-throughput screening of large databases. Through active learning loops, researchers can predict high-performance electrolyte components and inversely generate new molecular structures, while machine learning-accelerated molecular dynamics can reveal complex ion-pairing mechanisms and the dynamic evolution of ionic aggregates.

Notably, to improve the practical sustainability of AIBs, future research should extend beyond electrochemical performance to include holistic life cycle assessment (LCA). The current reliance on specific electrolyte components remains a major challenge to improving the overall sustainability of these systems. For instance, although AlCl_3_/[EMIm]Cl systems exhibit high ionic conductivity, the imidazolium-based cations (*e.g.*, [EMIm]^+^ and [BMIm]^+^) are generally considered environmentally persistent and have raised concerns regarding aquatic toxicity. Furthermore, the ubiquitous presence of AlCl_3_ introduces severe moisture sensitivity, and the hydrolysis of chloroaluminate species releases corrosive HCl gas, posing substantial risks to manufacturing personnel and ecosystem integrity. Similarly, chloride-free systems utilizing Al(OTf)_3_ or Al(TFSI)_3_ mitigate corrosivity but introduce fluorinated anions (OTF^−^, TFSI^−^). The strong C−F and S−F bonds in these compounds render them bio-accumulative and recalcitrant to biodegradation, raising long-term ecotoxicological concerns. Even in DES systems, although ligands such as urea or acetamide are inexpensive and readily available, their complexation with AlCl_3_ diminishes these advantages, producing electrolyte systems that still inherit the moisture sensitivity and corrosive characteristics associated with chloroaluminate species. Additionally, the high energy consumption required for the synthesis of high-purity fluorinated salts further increases the carbon footprint of these electrolyte systems. Therefore, current research has largely focused on improving electrochemical performance, whereas material hazards, life cycle impacts, and end-of-life management have received comparatively less attention. Future electrolyte design should prioritize the development of biodegradable ligands and more sustainable electrolyte chemistries without sacrificing anodic reversibility, thereby advancing both electrochemical performance and environmental sustainability.

In summary, room-temperature IL-based AIBs represent highly promising candidates for next-generation large-scale ESS. However, persistent challenges, such as the intrinsic passivation of aluminum anodes and the difficulty in achieving highly reversible aluminum deposition, continue to hinder their practical development. Over the past decade, fundamental research on this system has been considerably less extensive than that devoted to conventional organic battery systems and other multivalent metal batteries. Nevertheless, owing to the outstanding intrinsic advantages of aluminum metal, including its high theoretical capacity, excellent safety profile, and low cost, this technology remains of significant scientific and technological interest. Although certain electrolyte design principles may be transferable from other emerging battery chemistries, the development of tailored solutions is essential for aluminum batteries due to their unique ionic transport characteristics and complex SEI evolution mechanisms. As comprehensively reviewed herein, rationally designed modification strategies provide effective pathways to overcome these intrinsic limitations. Despite the substantial challenges that AIBs still face, we believe that with the growing global demand for efficient and sustainable energy storage, and the continued advancement of innovative electrolyte systems and interfacial stabilization strategies, AIBs are well positioned to meet future stringent requirements for low-cost, high-safety, and high-performance ESS applications.

## Conflicts of interest

There are no conflicts to declare.

## Data Availability

The data that support the findings of this study are available from the corresponding author upon reasonable request.
